# Multi-slice spatial transcriptome domain analysis with SpaDo

**DOI:** 10.1186/s13059-024-03213-x

**Published:** 2024-03-19

**Authors:** Bin Duan, Shaoqi Chen, Xiaojie Cheng, Qi Liu

**Affiliations:** 1 State Key Laboratory of Cardiology and Medical Innovation Center, Shanghai East Hospital, Frontier Science Center for Stem Cell Research, Bioinformatics Department, School of Life Sciences and Technology, Tongji University, Shanghai, 200092 China; 2 Key Laboratory of Spine and Spinal Cord Injury Repair and Regeneration (Tongji University), Ministry of Education, Orthopaedic Department of Tongji Hospital, Frontier Science Center for Stem Cell Research, Bioinformatics Department, School of Life Sciences and Technology, Tongji University, Shanghai, 200092 China; 3 Shanghai Research Institute for Intelligent Autonomous Systems, Shanghai, 201804 China; 4 Research Institute of Intelligent Computing, Zhejiang Lab, Hangzhou, 311121 China

**Keywords:** Spatial transcriptomics, Multiple slice analysis, Spatial domain detection

## Abstract

With the rapid advancements in spatial transcriptome sequencing, multiple tissue slices are now available, enabling the integration and interpretation of spatial cellular landscapes. Herein, we introduce SpaDo, a tool for multi-slice spatial domain analysis, including modules for multi-slice spatial domain detection, reference-based annotation, and multiple slice clustering at both single-cell and spot resolutions. We demonstrate SpaDo’s effectiveness with over 40 multi-slice spatial transcriptome datasets from 7 sequencing platforms. Our findings highlight SpaDo’s potential to reveal novel biological insights in multi-slice spatial transcriptomes.

**Supplementary Information:**

The online version contains supplementary material available at 10.1186/s13059-024-03213-x.

## Background

Spatially resolved transcriptomic technologies have revolutionized the detection of mRNA expression by preserving spatial information, thereby facilitating the exploration of biological functions at spatial level [1, 2]. In general, spatial transcriptomics technologies can be categorized into two types: high-throughput sequencing-based technologies [1, 3, 4] and fluorescence in situ hybridization (FISH)-based technologies [5], each exhibiting distinct advantages and limitations. Sequencing-based technologies provide high-throughput profiling of the whole transcriptomes while sacrifice spatial resolution, as they detect gene expression at multiple spatially defined sites called spots. FISH-based technologies such as MERFISH [6], seqFISH [7], seqFISH+ [8], osmFISH [9] et al., on the other hand, achieve single-cell resolution while exhibit lower throughput and limited transcript detection capabilities. Additionally, several in situ sequencing-based technologies, including STARmap [10] and FISSEQ [11], achieve single-cell resolution, however, with lower throughput. Recently, Stereo-seq has emerged as a technology capable of achieving subcellular resolution while also maintaining high throughput [12].

Despite the development of various spatially resolved transcriptome technologies, the analysis of spatial transcriptomics data remains a significant challenge, particularly in the context of spatial domain analysis. Spatial domains refer to specific regions in space that consist of multiple cells and are often associated with the tissue’s anatomical structure and specific functions [13]. These spatial domains can be considered as the fundamental functional units that contain spatial information for downstream analysis. Currently, several methods have been developed for spatial domain detection, which can be categorized into three groups: (1) traditional domain detection methods, including Seurat [14] and Scanpy [15] et al., which do not explicitly consider spatial information. These methods are often used as baselines for domain detections. (2) Statistical model-based methods, such as BayesSpace [16] et al., are developed based on the simple assumption that spatially adjacent spots are more likely to exhibit similar gene expression patterns. However, they are not designed specifically to handle single-cell spatial transcriptome. (3) Graph neural network-based methods, including SpaGCN [17], SEDR [18], STAGATE [19] et al., assume that the gene expression information of each spot can be reconstructed using its neighboring information. Typically, a low-dimensional embedding of each spot, containing spatial information, is obtained. Nonetheless, challenges like relatively high computational complexity or limited interpretability may exist.

In addition, although existing methods can detect spatial domains within a single tissue slice, they cannot directly handle multi-slice spatial domain analysis for multiple tissue slices in general. With the rapid advancement of spatial transcriptomes, there is an increasing accumulation of multiple tissue slices that can be integrated to unravel spatial cellular landscapes. For instance, the integration of multi-slice spatial transcriptomic data identified specific cell types with spatial dependencies in the diseased state of myocardial infarction, shedding light on new pathogenic mechanisms and novel therapeutic options [20]. In addition, the analysis across multiple slices revealed that tertiary lymphoid structures (TLS), which are organized immune cell groups found in nonlymphoid tissues and are often associated with improved tumor prognosis, have exhibited stable and consistent cell type composition [21]. Therefore, multi-slice spatial transcriptome domain analysis is fundamental in dealing with the accumulating multi-slice spatial transcriptomic data, despite the significant challenges it presents. It is worth noting that several computational methods, such as PASTE [22] and SLAT [23], have been developed for pairwise alignment of slices. However, these methods are primarily focused on aligning single cells or spots. They cannot be applied directly to analyze spatial domains across multiple slices, thereby limiting their applications.

To this end, we propose SpaDo (multi-slice spatial transcriptome domain analysis) for multi-slices spatial transcriptome analysis at both single-cell and spot resolution. Specifically, SpaDo contains three functional modules: multi-slice spatial domain detection, reference-based spatial domain annotation, and multi-slice clustering analysis. SpaDo shows several key advantages, including good interpretability, robustness, and tolerance to noise and batch effects.

The superiority of SpaDo is demonstrated by a comprehensive investigation on over 40 sets of multi-slice spatial transcriptomic data obtained from 7 different spatial transcriptome sequencing platforms, including osmFISH, seqFISH + , STARmap, MERFISH, 10 × Visium, old ST, and Slide-seqV2 [4] (Additional file 1: Table S1). The results of our study highlight the significant potential of SpaDo to gain novel biological insights from multi-slice spatial transcriptomes.

## Results

### Overview of SpaDo

**Fig. 1:**
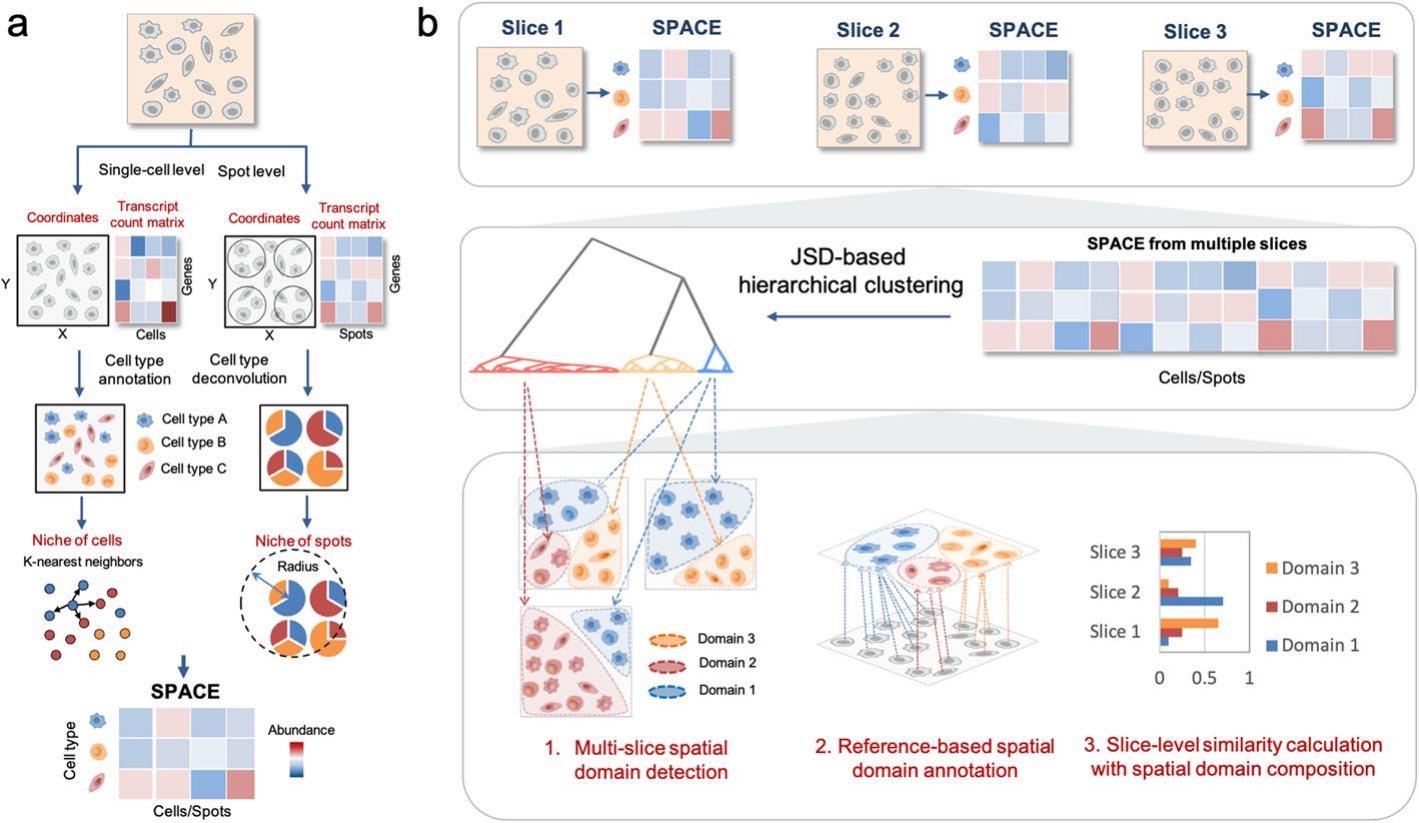
Workflow of SpaDo. **a** Calculating SPACE for both single-cell and spot resolution spatial transcriptomic data. SPACE SPatially Adjacent Cell type Embedding. **b** Three functions involved in multi-slice spatial domain analysis: multi-slice domain detection, reference-based spatial domain annotation, and multi-slice clustering analysis by consideration of spatial domain composition. JSD Jensen–Shannon divergence

SpaDo is a comprehensive computational framework for multi-slice spatial domain analysis, including four main components (Fig. 1): (1) cell type annotation, (2) calculation of SPatially Adjacent Cell type Embedding (SPACE), (3) Jensen–Shannon divergence (JSD)-based hierarchical clustering, and finally (4) multi-slice spatial domain analysis.

SpaDo firstly requires proper cell type annotations for each slice. Depending on the resolution of the spatial transcriptomic data (either single-cell or spot resolution), different strategies are adopted (Fig. 1a and see “Methods”). For single-cell resolution spatial transcriptomics data, reference-based annotation methods [24, 25] or clustering methods such as Seurat v4 [14] are recommended. For spot resolution spatial transcriptomics data, Cell2location [26] is used as spot deconvolution method for SpaDo.

Secondly, SpaDo calculates SPatially Adjacent Cell type Embedding (SPACE). SpaDo initiates the process by determining the local niche of each cell/spot through a search of k-nearest neighbors in the case of single-cell resolution spatial transcriptomics data or neighbors within a specific radius for spot resolution spatial transcriptomics data. SPACE is subsequently derived by fusing cell type annotations with the niche information of each cell/spot, thereby integrating gene expression and spatial information.

Thirdly, SpaDo has the capability to identify spatial domains across all spots/cells from multiple slices through hierarchical clustering of SPACE, based on Jensen–Shannon divergence, across all spots/cells from multiple slices. Consequently, the spatial domains identified by SpaDo are derived from a combination of multiple slices rather than being limited to a single slice. By tracing back and mapping these detected spatial domains to each individual slice, SpaDo enables the alignment of spatial domains across multiple slices, thereby facilitating multi-slice spatial domain analysis (Fig. 1b and see “Methods”).

Finally, SpaDo enables multi-slice spatial domain analysis, including: (1) multi-slice spatial domain detection: SpaDo detects consistent spatial domains across multiple slices; (2) reference-based spatial domain annotation: SpaDo leverages spatial references, which are spatial transcriptomic data with domain annotations, to annotate new sequenced spatial transcriptomic data. Specifically, spatial domain labels in reference are assigned to the query cells based on the minimum distance, and (3) multi-slice clustering analysis: SpaDo calculates slice-level similarity using spatial domain composition (see “Methods”) and performs clustering analysis at the slice level. This function is particularly useful for analyzing spatial transcriptomics data with multiple time points or varying conditions.

Particularly, we would like to highlight the advantages of SPACE embedding comparing to other complex GNN-based spatial embedding methods like SEDR, SpaGCN, and STAGATE: (1) SPACE is highly interpretable, aligning well with the biological nature of spatial domains, which often encompass specific cell types with functional relationships [13, 21, 27–29]. (2) SPACE effectively addresses batch effects, as it is designed based on the spatial relationships of cell types, ensuring consistency across slices through various automated cell type annotation strategies [24–26, 30] and clustering methods [14, 15]. As a result, SPACE is naturally suited for integration between multiple slices without the need for additional batch correction. (3) SPACE primarily relies on cell type annotations rather than detailed spatial information. The differences between spatial domains are apparent and do not require fine details, as supported by two recent studies [31, 32]. This characteristic makes SPACE tolerant to noise and robust to diverse variations in spatial transcriptomics data.

### Evaluation of SpaDo for single-slice spatial domain detection

**Fig. 2:**
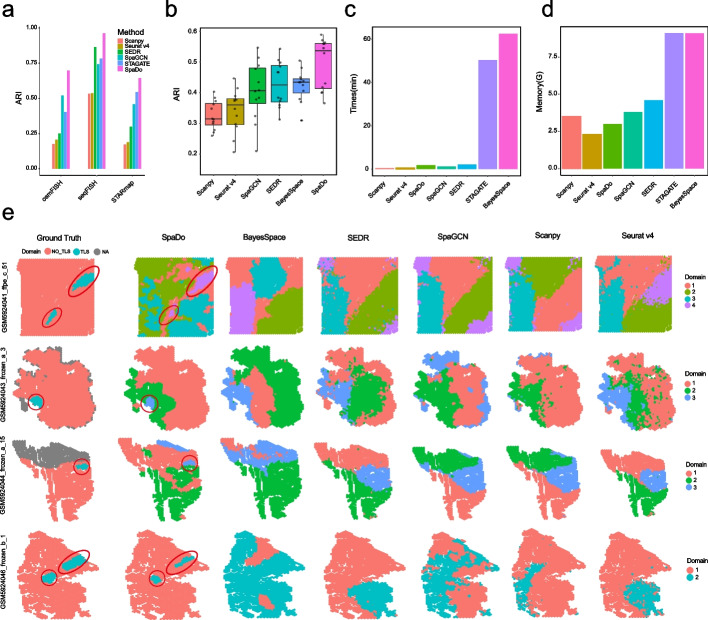
SpaDo outperforms existing single-slice spatial domain detection methods. **a** Performance of SpaDo and other methods (Scanpy, Seurat v4, SEDR, SpaGCN, and STAGATE) on three single-cell spatial transcriptomic datasets. ARI adjust rand index. **b** Performance of SpaDo and other methods (Scanpy, Seurat v4, SpaGCN, SEDR, and BayesSpace) on 12 spot resolution spatial transcriptomic datasets. **c** Single CPU (SpaDo, Scanpy, Seurat v4, SpaDo, BayesSpace) and single GPU (SpaGCN, SEDR, STAGATE) runtime when tested in DLPFC_151673 data (3639 spots). **d** Single CPU (SpaDo, Scanpy, Seurat v4, SpaDo, BayesSpace) and single GPU (SpaGCN, SEDR, STAGATE) memory usage when tested in DLPFC_151673 data. **e** Visualization of TLS-like domain detection of SpaDo and other methods (BayesSpace, SEDR, SpaGCN, Scanpy, and Seurat v4) on 4 RCC spatial transcriptomic datasets

We firstly conducted a comprehensive evaluation of SpaDo against other single-slice-based spatial domain detection methods for both single-cell and spot resolution spatial transcriptome (Fig. 2), which serves as the foundation for multi-slice spatial domain analysis.

The spatial domains detected by SpaDo form a multi-resolution schema hierarchically, enabling the characterization of spatial structures dynamically with different resolutions. In order to distinguish the spatial domains under different resolutions, we adopt the following naming scheme for the spatial domains by assigning each domain in a specific resolution with a term ID “Domain_N_1__N_2_”, where N_1_ represents the resolution level (i.e., the number of detected domains, the larger the higher resolution) and N_2_ represents the ID of specific domain. For illustration, we elucidate the naming scheme with DLPFC_151673 (10 × Visium) data from the dorsolateral prefrontal cortex (DLPFC) datasets [33], as detailed in Additional file 1: Fig. S1. Furthermore, we present the results of multi-resolution spatial domain detection for osmFISH (Additional file 1: Fig. S2) and STARmap data (Additional file 1: Fig. S3).

To illustrate SpaDo’s superior performance in single-slice spatial domain detection, we conducted a comparative analysis with well-established single-slice spatial domain detection methods, including Seurat v4 [14], Scanpy [15], SEDR [18], SpaGCN [17], STAGATE [19], and BayesSpace [16]. Our evaluation covered three key perspectives: (1) spatial domain detection on single-cell spatial transcriptomics data, (2) spatial domain detection on spot resolution spatial transcriptomics data, and (3) TLS-like domain detection.

For the first test (Fig. 2a), we can see that SpaDo outperformed Seurat v4, Scanpy, SEDR, SpaGCN, and STAGATE across three single-cell spatial transcriptomic datasets from three different platforms: osmFISH [27], STARmap [10], and seqFISH+ [8], each exhibiting different levels of complexity in domain structures (Fig. 2a and Additional file 1: Figs. S4-S6). We excluded BayesSpace in this test as it is specifically designed for spot resolution spatial transcriptome.

For the second test (Fig. 2b), SpaDo outperformed Seurat v4, Scanpy, SEDR, SpaGCN, and BayesSpace on 12 spot resolution dorsolateral prefrontal cortex (DLPFC) datasets [33]. STAGATE was excluded from this test due to its instability and occasional failure with spot resolution data. In addition, using two DLPFC datasets (DLPFC_151675 and DLPFC_151676) as examples, we demonstrated SpaDo’s ability to significantly improve its performance by integrating multiple slices (Additional file 1: Fig. S7). This underscored SpaDo’s adaptability and effectiveness in optimizing results, especially in challenging dataset scenarios.

For the third test (Fig. 2e), we evaluate four renal cell cancer (RCC) slices [34] with well-annotated tertiary lymphoid structures (TLS) regions. TLS is a widely recognized spatial domain with a relatively consistent cell type composition, primarily consisting of T cells and B cells. In this test, we utilized the minimum domain number at which SpaDo can detect TLS-like domains. This choice was made to highlight SpaDo’s maximum sensitivity in comparison to other methods. As shown in Fig. 2e, SpaDo outperformed other methods by accurately detecting TLS-like domains that aligned well with the manually annotated labels. We also evaluated the effect of varying the number of domains and found that SpaDo consistently outperformed other methods, even when selecting a larger number of domains (Additional file 1: Fig. S8). Furthermore, SpaDo is user-friendly, demonstrating both time efficiency (Fig. 2c) and memory usage efficiency (Fig. 2c–d).

### The validation of robustness of SpaDo

**Fig. 3:**
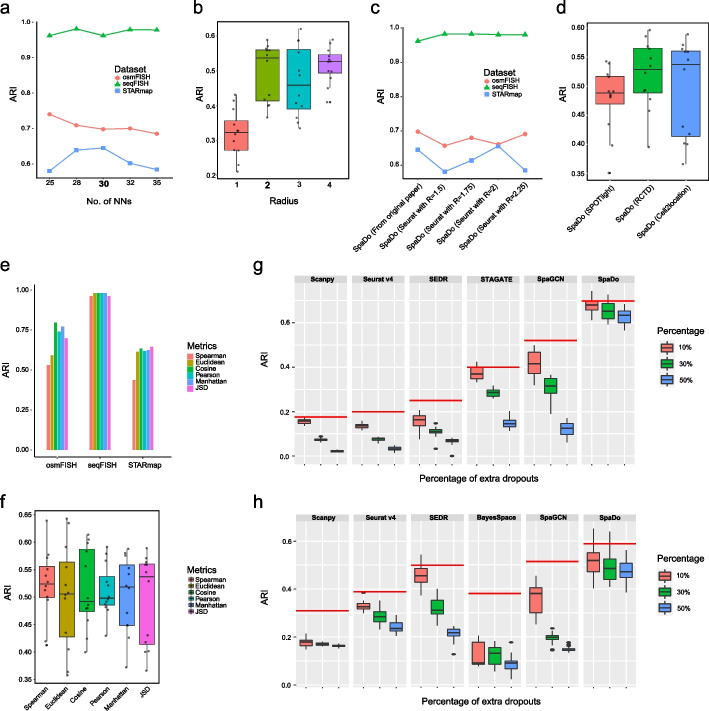
The validation of robustness of SpaDo. **a** Performance of SpaDo with different numbers of nearest neighbors on three single-cell spatial transcriptomic datasets. No. of NNs: number of nearest neighbors. **b** Performance of SpaDo with different radius. **c** Performance of SpaDo when using cell type annotation from original paper or Seurat v4 with different value of parameter “Resolution” on osmFISH, seqFISH, and STARmap datasets. R resolution. **d** Performance of SpaDo when using different spot deconvolution methods. **e** Performance of SpaDo when using different distance metrics on three single-cell spatial transcriptome datasets. **f** Performance of SpaDo when using 12 DLPFC datasets. **g** Performance of SpaDo with increased dropout rates on osmFISH dataset. The red line represents the original performance of each method. **h** Performance of SpaDo with increased dropout rates on DLPFC_151673 dataset. Percentage of extra dropouts is shown on the right of the plots. The red line represents the original performance of each method

The robustness of methods for analyzing spatial transcriptomics data is crucial, given the inherent noise in such data. Therefore, we conducted a comprehensive analysis to assess SpaDo’s robustness from the following four different aspects (Fig. 3).

Firstly, we evaluated the robustness of two key parameters in SpaDo: the number of nearest neighbors for single-cell resolution spatial transcriptomic data (Fig. 3a) and the radius sizes for spot resolution spatial transcriptomic data (Fig. 3b). We found that SpaDo maintains robustness across varying numbers of nearest neighbors (Fig. 3a) and a range of radius sizes (Fig. 3b). It is worth noting that when Radius = 1, SpaDo may not fully leverage spatial information unless the spot radius for spatial transcriptome sequencing methods is very large, such as old ST, so it is lower than other radius value significantly, which is included here solely as a baseline.

Secondly, SpaDo relies on accurate cell type annotations for both single-cell and spot resolution spatial transcriptomics data. In this study, SpaDo employed Seurat v4 for single-cell spatial transcriptomics data and Cell2location [26] for spot resolution data to obtain these annotations. The Cell2location was selected based on its strong performance in a third-party benchmarking paper [35]. Furthermore, we validated the robustness of SpaDo to Seurat v4 parameters for cell type annotation across three single-cell spatial transcriptomics datasets (Fig. 3c). We also investigated the application of other widely used spot deconvolution methods, including SPOTlight [36] and RCTD [37], for 12 spot resolution DLPFC slices (Fig. 3d). We can see that SpaDo is robust to spot deconvolution methods, with Cell2location achieving the highest median.

Third, we utilized Jensen–Shannon divergence (JSD) to calculate the distance for SpaDo’s SPACE of each spot/cell. To assess SpaDo’s robustness to different distance metrics, we evaluated its performance using commonly employed metrics like Spearman correlation, Pearson coefficient, Cosine distance, Euclidean distance, Manhattan distance, and JSD for both single-cell (Fig. 3e and see “Methods”) and spot resolution spatial transcriptomics data (Fig. 3f and see “Methods”). SpaDo consistently demonstrated robustness across these different distance metrics, with JSD delivering the highest median.

Lastly, given the inherent noise in spatial transcriptomics data, we assessed SpaDo and other existing methods’ sensitivity to sequencing depth and dropouts. Taking osmFISH (single-cell resolution) and DLPFC_151673 (spot resolution) data as examples, we artificially increased dropout rate by randomly setting 10%, 30%, and 50% of the nonzero expression values to zero. We observed that SpaDo exhibited tolerance to higher dropout rates, while other methods were notably affected (Fig. 3g, h and see “Methods”).

### SpaDo effectively mitigates batch effects in multi-slice integration

Most existing spatial transcriptome analysis methods are limited to single-slice spatial domain analysis, as they are unable to integrate gene expression and spatial information across multiple slices. However, with the advancement of spatial transcriptomics, it has become possible to obtain multiple slices from the same tissue and integrate them to gain novel biological insights [20, 21].

Notably, one of the most significant challenges lies batch effect during the integration process. As mentioned earlier, SpaDo utilizes SPACE to integrate gene expression and spatial information. SPACE is designed to leverage the spatial relationships among cell types, which ensures consistency across slices through various automated cell type annotation strategies [24, 25, 30] and spot deconvolution methods [14, 15]. Therefore, SpaDo is theoretically tolerance to batch effects.

**Fig. 4:**
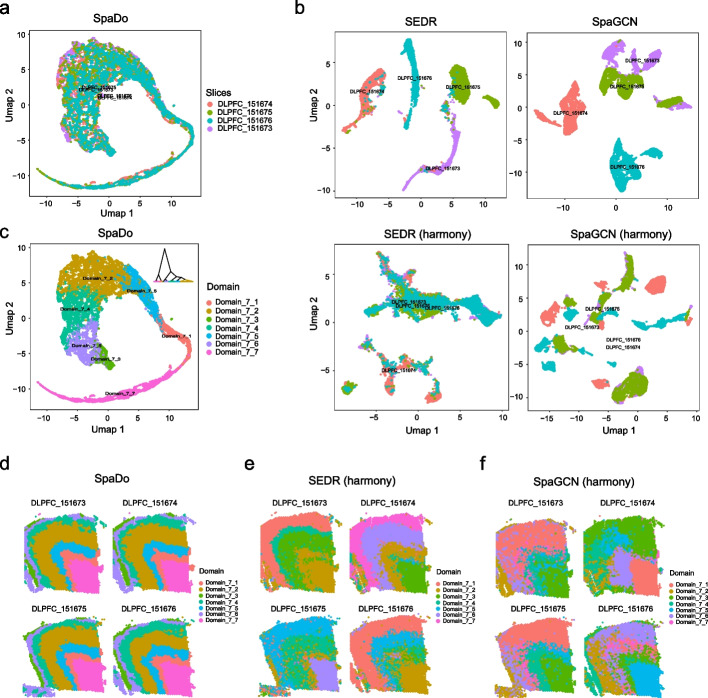
The batch effects evaluation of SpaDo and other existing methods for multi-slice domain detection. **a** Umap of SpaDo on four DLPFC slices (colored by slices). **b** Umap of SEDR and SpaGCN with and without harmony on four DLPFC slices (colored by slices). **c** Umap and hierarchical clustering result of SpaDo on four DLPFC slices (colored by detected spatial domains). **d** Locations of spatial domains detected by SpaDo on four DLPFC slices. **e** Locations of spatial domains detected by SEDR incorporated with harmony on four DLPFC slices. **f** Locations of spatial domains detected by SpaGCN incorporated with harmony on four DLPFC slices

To further prove this point, we made comprehensive comparisons between SpaDo and other existing methods for addressing batch effects on both single-cell and spot resolution spatial transcriptomics data (Fig. 4 and Additional file 1: Fig. S9). For spot resolution spatial transcriptomics, we tested four DLPFC slices, which belong the same sample with the same seven layers. We compared SPACE utilized in SpaDo (Fig. 4a), embeddings obtained by SEDR, and SpaGCN with and without harmony [38] to correct batch effects (Fig. 4c). The results demonstrate that SpaDo’s spatial embeddings (SPACEs) effectively mitigate batch effects across multiple slices. In contrast, the embeddings from SEDR and SpaGCN, even when combined with harmony [38], do not align well among multiple slices. Furthermore, we performed multi-slice domain detection with SpaDo (Fig. 4c, d). Then, for embeddings obtained by SEDR and SpaGCN combined harmony, we adopted similar strategy to perform multi-slice domain detection (Fig. 4e, f and see “Methods”). We can see that only SpaDo obtained consistent domain results between 4 DLPFC slices (Fig. 4d–f). We obtained the similar conclusions on 3 MERFISH data (Additional file 1: Fig. S9).

We clarify this observation by emphasizing that embeddings generated through alternative methods, such as SEDR, SpaGCN, and STAGATE, are restricted to distinct training spaces within individual data slices. As a result, embeddings from different slices produced by these methods do not align, even when applying batch correction techniques like harmony. In contrast, SPACE is inherently grounded in the spatial distribution of cell types, a feature that remains uniform across diverse slices. The good interpretability of SPACE in representing spatial domains, combined with this consistency, forms the foundation for its superior performance.

### SpaDo enables multi-slice spatial domain detection

We illustrated the utility of SpaDo to detect spatial domains that can be comparable across multiple slices, which is crucial for studying shared spatial function across slices.

**Fig. 5:**
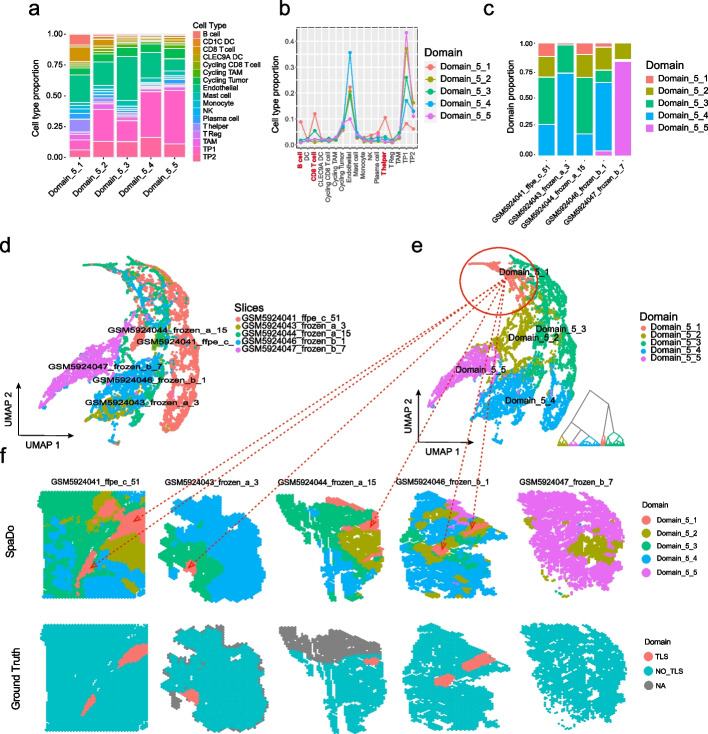
SpaDo enables consensus spatial domain detection across multiple slices. **a** Cell type proportion of each detected domain. **b** Comparison of cell type proportion in each detected spatial domain. **c** Proportion of detected spatial domains for each RCC slice. **d** Umap and hierarchical clustering result of SPACE on five RCC slices (colored by slices). **e** Umap and hierarchical clustering result of SPACE on five RCC slices (colored by detected spatial domains). **f** Location of detected spatial domains against manually annotated TLS labels in all five RCC slices

Specifically, we performed TLS-like domain detection across multiple slices (Fig. 5).

We used five RCC slices with manually annotated TLS regions, four of which contained one or two TLS regions, while the remaining slice with no TLS region served as a negative control [34]. In this test, SpaDo successfully detected five spatial domains with default spatial domain number selection method (Fig. 5e and see “Methods”). In addition, SpaDo also provided the exact proportion of each cell type within each detected domain, demonstrating its good interpretability (Fig. 5a). Notably, among the five spatial domains, “Domain_5_1” exhibited significant enrichment of B cells, CD8 T cells, and T helper cells, distinguishing it from the other four spatial domains (Fig. 5b). Furthermore, “Domain_5_1” was detected as a consensus domain showed in all four TLS positive slices but not in the negative control slice (Fig. 5c). These findings indicate that “Domain_5_1” represents a common TLS-like spatial domain associated with important immune function. The locations of “Domain_5_1” in the four slices were consistent with the manually annotated TLS labels (Fig. 5f), further confirming the capability of SpaDo to detect consensus spatial domains across multiple slices. These consensus spatial domains can be potential spatial markers. Finally, similar results were obtained even when the spatial domain number was set to 3, demonstrating the strong robustness and high sensitivity of SpaDo to detect TLS-like spatial domain (Additional file 1: Fig. S10).

### SpaDo enables reference-based spatial domain annotation

The annotation of spatial domains is an important task while primarily done manually, which is laborious and time-consuming. As spatial transcriptomic datasets with manually annotated spatial domains are becoming more prevalent, SpaDo can be used to annotate spatial domain automatically. Specifically, SpaDo leverages these datasets, referred as spatial references with spatial domain labels, to annotate newly sequenced spatial transcriptomes. This strategy is conceptually similar to the popular tools like BLAST [39] for sequence alignment or our previous strategy scLearn et al. for automatic cell type annotations using single-cell transcriptomic references [24, 25, 30]. In the case of SpaDo, it performs a search by measuring the distance between the SPACE of the query cells and that of the centroid of each spatial domain in the spatial reference. Then, spatial domain labels in reference are assigned to the query cells based on the minimum distance (see “Methods”).

**Fig. 6:**
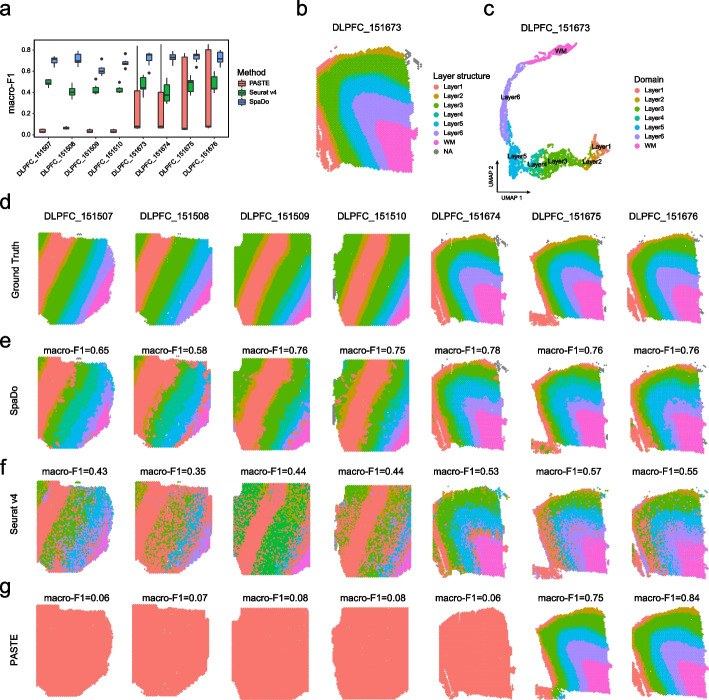
Benchmarking SpaDo with other methods for automatically annotating spatial domains. **a** macro-F1 of SpaDo, Seurat v4, and PASTE on eight DLPFC datasets. **b** Locations of manually annotated spatial domains in dataset “DLPFC_151673”. **c** Umap of SPatially Adjacent Cell type Embedding calculated by SpaDo (colored by manually annotated spatial domains). **d** Manually annotated spatial domains of eight DLPFC datasets. **e** Locations of spatial domains annotated by SpaDo. **f** Locations of spatial domains annotated by Seurat v4. **g** Locations of spatial domains annotated by PASTE

Currently, there are only a few methods available for the automatic annotation of spatial domains, such as Seurat v4 and PASTE [22]. Seurat v4 annotates spatial domains using a similar strategy of SpaDo. However, Seurat v4 calculates the spatial domain centroid based on gene expression solely without spatial information. PASTE annotates spatial domains by performing pairwise alignment of slices to find the optimal probabilistic mapping between spots in one slice and spots in the other slice. However, PASTE is limited to adjacent slices thus unsuitable for diverse slices. To demonstrate the superiority of SpaDo in this task, we tested eight commonly used DLPFC datasets [33] for spatial domain annotations. Each dataset was treated as a reference or query data, respectively, resulting in a total of 56 dataset pairs (permutation ${A}_{8}^{2}$ = 56) (Fig. 6a and see “Methods”). As a result, it is clearly shown that SpaDo achieved a much higher macro-F1 score compared to Seurat v4 and PASTE (Fig. 6a). Additionally, we illustrated these results using “DLPFC_151673” as the spatial reference and the remaining seven DLPFC datasets as queries. The UMAP visualization of SPACE calculated by SpaDo for “DLPFC_151673” was highly consistent with the manually annotated spatial domain annotations (Fig. 6b, c). Furthermore, SpaDo outperformed Seurat v4 in terms of annotation accuracy for all query datasets (Fig. 6d, f). PASTE, on the other hand, almost failed in five out of seven datasets, predicting all spots as belonging to the same spatial domain (Fig. 6g).

### SpaDo enables multi-slice clustering analysis

**Fig. 7:**
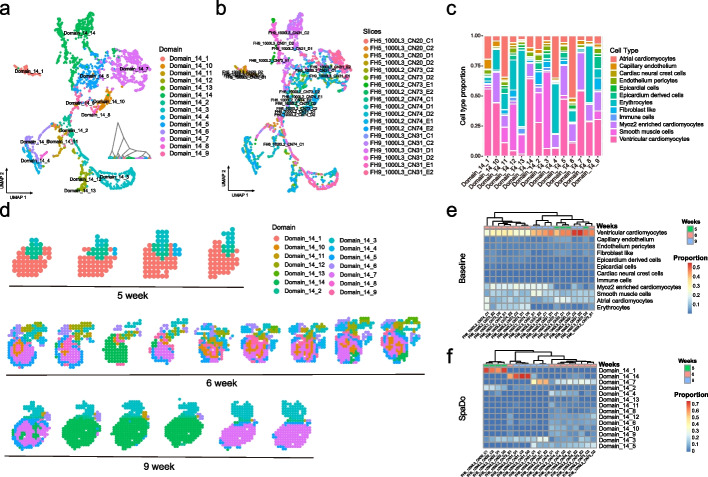
SpaDo enables multi-slice clustering analysis. **a** Umap and hierarchical clustering result of SPACE on 19 human heart slices (colored by detected spatial domains). SPACE SPatially Adjacent Cell type Embedding. **b** Umap of SPACE on 19 human heart slices (colored by slices). **c** Cell type proportion of each detected domain. **d** Location of each detected domain in 19 slices. **e** Heatmap of clustering results of 19 slices using cell type composition as baseline. **f** Heatmap of clustering results of 19 slices using spatial domain composition detected by SpaDo

Multi-slice clustering analysis plays a crucial role in studying spatial heterogeneity alongside developmental status [40, 41]. The key idea to perform multi-slice clustering analysis lies in calculating similarity of different slices. Traditionally, the global similarity of multiple slices is measured based on the assumption that similar slices have a similar cell type composition, without considering spatial information. However, SpaDo takes a different perspective by assuming that if two slices are similar, their spatial domain compositions (Figs. 1b and 7f, and see “Methods”) are also similar, and vice versa. This enables SpaDo to perform multi-slice clustering analysis properly.

As a result, we conducted a comparison using three spatial transcriptomic studies [40–42] to evaluate the performance of SpaDo for multi-slice clustering analysis. The first study focused on constructing a spatiotemporal cell atlas of the developing human heart, utilizing 19 spatial transcriptomic (ST) slices from the developing human heart at three developmental stages, i.e., 5, 6, and 9 post-conception weeks (PCW) (Fig. 7). We initially performed multi-slice spatial domain detection using SpaDo (Fig. 7a). The datasets with the same time points tended to cluster together, indicating that SpaDo effectively captured the underline information of each slice without being affected by batch effects (Fig. 7b). It was observed that slices from the same time points exhibited similar spatial domain compositions (Fig. 7d). Furthermore, when compared to the baseline method (calculating slice similarity with cell type composition), SpaDo demonstrated more consistent clustering results (Fig. 7e, f, and see “Methods”). Importantly, this improved performance was found to be robust across different selected domain numbers (Additional file 1: Fig. S11). Similar results were obtained in the other two datasets, i.e., the developing chicken heart dataset [42] (Additional file 1: Figs. S12 and 13) and the human cortical organoid dataset [41] (Additional file 1: Fig. S14). These findings highlight the capability of SpaDo to efficiently integrate spatial information to measure the global similarity of multiple slices in multi-slice clustering.

## Discussion

With the advancement of spatial transcriptomes, multiple tissue slices are increasingly accumulating and can be further integrated to uncover new insights into transcriptomic and cellular landscapes. However, the challenge lies in effectively integrating gene expression and spatial information in a manner that is both interpretable and comparable across multiple slices. Current strategies have been limited to single-slice domain analysis with relatively high computational complexity and limited interpretability. Therefore, we propose SpaDo as an efficient framework designed to facilitate multi-slice spatial domain analysis at both single-cell and spot resolution.

SpaDo performs spatial transcriptomics analysis with three key applications: multi-slice spatial domain detection, reference-based spatial domain annotation, and multi-slice clustering analysis. With examination of over 40 diverse spatial slices from various biological contexts and sequencing platforms, we proved that SpaDo is robust to different parameters and tolerant to noise (Fig. 3). In addition, SpaDo effectively addresses batch effects without additional correction. In summary, SpaDo greatly enhances the analysis of spatial transcriptomics data, especially in scenarios involving multi-slice spatial domains.

The key innovation of SpaDo lies in its design of a simple yet effective embedding called SPACE. By combining cell type annotation with spatial niche, SPACE successfully integrates gene expression and spatial information across multiple slices, demonstrating tolerance to high noise and batch effects (Figs. 3 and 4). We highlight three main reasons why SPACE outperforms other complex spatial embedding methods like SEDR, SpaGCN, and STAGATE: (1) SPACE demonstrates good interpretability by aligning well with the biological characteristics of spatial domains. (2) SPACE effectively addresses batch effects. (3) Differences between distinct spatial domains are relatively obvious and do not require particularly fine spatial information to distinguish them [31]. Therefore, although SPACE may smooth features and reduce tissue heterogeneity, it still performs well in multi-slice spatial domain analysis. In our study, we did not observe that SPACE simplifies spatial structures (Figs. S4 and S6, and Fig. 2e). We speculate that three reasons may exist here. Firstly, while there may be a loss of spatial information at the individual cell level, the essential spatial information—specifically, the composition of cell types throughout the entire spatial domain—is effectively preserved. Secondly, the inherent noise resistance of smooth operation aids in eliminating noise from spatial transcriptomic data by using SPACE embedding. Lastly, recent studies [31, 32] support that the discernible distinctions between spatial domains do not necessitate intricate details. In summary, SPACE maintains a delicate balance between noise tolerance and feature smoothing in spatial domain detection, making it well-suited for the integration of multiple slices.

SpaDo has three potential limitations. Firstly, it depends on cell type annotation methods like Seurat v4 and Cell2location. Nevertheless, even in scenarios where cell type annotation methods exhibit slightly suboptimal performance, SpaDo continues to demonstrate relatively favorable outcomes (Fig. 3c, d). Secondly, while we have demonstrated that SPACE, designed by SpaDo, is well-suited for spatial domain analysis, it may tend to smooth features and reduce tissue heterogeneity. Therefore, caution should be exercised when applying SPACE to analyses beyond the spatial domain. Thirdly, SpaDo specifically focus on the spatial domain analysis of multiple slices, which maybe not suitable for cell-level 3D tissue reconstruction.

While our study primarily focuses on applying SpaDo to spatial transcriptomic data, it is noteworthy that SpaDo holds the potential for extension into multimodality spatial data analysis. This extension could be particularly valuable if corresponding cell types across different omics datasets are identifiable. Notably, advancements in spatial epigenomics [43, 44] and technologies like slide-DNA-seq [45] present exciting opportunities for integrating epigenetic and DNA information into spatial analyses. As spatial DNA-seq technologies like slide-DNA-seq continue to evolve, they offer the potential to decipher more accurate tumor evolution patterns [46]. SpaDo, in turn, is poised to leverage these developments and combine spatial multimodal data from multiple slices. This integration holds the promise of uncovering new tumor markers, including both consistent and differential tumor evolution patterns, thereby contributing to a deeper understanding of spatial biology.

## Conclusion

In summary, SpaDo stands out as a pioneering framework for multi-slice spatial domain analysis in spatial transcriptomics. Its superior performance in detecting multi-slice spatial domains, providing reference-based spatial domain annotation, and conducting multi-slice clustering analysis addresses the limitations of single-slice domain analysis. The SPACE embedding ensures good interpretability, strong robustness, and high noise tolerance, making SpaDo a valuable spatial transcriptomics analysis tool for researchers.

## Methods

### Data description

SpaDo is designed to be compatible with all spatial transcriptomic sequencing technologies and platforms. In this study, we specifically tested its performance on the osmFISH, STARmap, seqFISH + , MERFISH, 10 × Visium, ST, and Slide-seq V2 platforms (Additional file 1: Table S1). Notably, the DLPFC dataset [33] includes 12 human DLPFC slices sampled from three individuals. The DLPFC layers and white matter (WM) were manually annotated by the original study. To obtain the cell type abundance of the above 12 DLPFC slices, we performed spot deconvolution using Cell2location [26] with a single-cell transcriptomic data [47] of DLPFC as reference.

The MERFISH dataset [48] used in our study comprised three samples with Animal_IDs 31, 32, and 33. These samples were characterized by a Bregma value of 0.16 and a specific behavioral trait described as “Aggression to adult”.

The RCC dataset [34] used in our study includes five RCC slices (10 × Visium): “GSM5924041_ffpe_c_51”, “GSM5924043_frozen_a_3”, “GSM5924044_frozen_a_15”, “GSM5924046_frozen_b_1”, and “GSM5924047_frozen_b_7”. Among these, the first four slices contain one or two TLS regions, while the last slice without TLS region is taken as negative control. We performed spot deconvolution of the above 5 RCC slices using Cell2location with single-cell transcriptomic data [49] (P76 and P90) of RCC as the reference, and the annotations of cell subtypes from the original study were merged to 17 main cell types.

The human heart dataset [40] used in our study consists of 19 slices (ST), representing the developing human heart at three developmental stages in the first trimester: 5, 6, and 9 post-conception weeks (PCW). We used single-cell transcriptomic data from the original study as a reference to obtain the cell type deconvolution results with Cell2location.

The chicken heart dataset [42] used in our study consists of 11 slices (10 × Visium) obtained from the early to late four-chambered heart stage: 4, 7, 10, and 14 days. We used single-cell transcriptomic data from the original study as a reference to obtain the cell type deconvolution results with Cell2location.

The organoid dataset [41] used in our study comprises 10 slices (Slide-seq V2) obtained from the developing human cortical organoid at 1, 2, and 3 months. Given the high resolution of Slide-seq V2 (Each spot has a diameter of 10 μm and contains about 1–3 single cells), we analyzed the organoid dataset as single-cell resolution spatial transcriptomic data. Cell type labels were obtained from the original study.

### Data preprocessing

We applied different normalization methods depending on the spatial transcriptomic platform used for data generation. For datasets generated from osmFISH, STARmap, and MERFISH platforms, we followed the normalization methods recommended by their respective original studies. This involved dividing the gene counts per cell by the total counts per cell, followed by a log transformation (log(1 + normalized counts)). For datasets obtained from other platforms, including seqFISH + , 10 × Visium, ST, and Slide-seq V2, we performed the standard normalization procedure using the Seurat package. This involved normalizing the gene expression measurements for each cell/spot by the total expression, multiplying the result by a scaling factor of 10,000, and finally applying a log transformation (log(1 + normalized counts)).

### Cell type annotation

SpaDo employs distinct strategies for cell type annotation depending on the resolution, whether it is at the single-cell or spot level.

For single-cell resolution spatial transcriptomics data, in our study, we utilized cell type labels from the original studies. In cases where these labels were unavailable, we employed Seurat v4, selecting the top 2000 highly variable genes with default parameters and obtaining cell type annotation results using the parameter “resolution = 2”. Importantly, the robustness of SpaDo to the “resolution” parameter of Seurat v4 was demonstrated in our analysis (Fig. 3c). It is important to note that for multi-slice single-cell resolution transcriptomic data, Seurat must be applied to the entire multi-slice gene expression profile to ensure consistent cell type annotation results.

On the other hand, for spot resolution spatial transcriptomics data, in our study, SpaDo specifically utilized Cell2location [26] to obtain spot annotations. The robustness of SpaDo to other spot deconvolution methods, such as RCTD and SPOTlight, was also demonstrated (Fig. 3d). It is important to highlight that, for multi-slice spot resolution transcriptomic data, Cell2location should be applied to the entire multi-slice dataset using the same single-cell reference to ensure consistent spot deconvolution results.

### Calculating SPatially Adjacent Cell type Embedding

SpaDo employed two distinct strategies to calculate SPatial Adjacent Cell type Embedding (SPACE) for single-cell and spot resolution spatial transcriptomic data, respectively. For single-cell spatial transcriptomic data, the K-nearest neighbors (KNN) method was used to identify the adjacent cells of each cell because KNN is able to take full advantages of density information. Then, SpaDo calculated the cell type proportion of these adjacent cells, obtaining the SPACE for each cell. For spot resolution spatial transcriptomic data, SpaDo obtained adjacent neighbors of each spot by searching within a specified radius as spot is distributed evenly. Then, SpaDo calculated the cell type proportion using the deconvolution results of these adjacent spots.

In multi-slice domain detection, we initially generated a SPACE for each slice. Given the consistent cell type annotations across all slices, meaning they share the same embedding space, the SPACEs of cells/spots from different slices became comparable. SpaDo achieved this by concatenating each individual SPACE, thereby obtaining a unified SPACE representation for multiple slices.

### Spatial domain detection

In this study, spatial domains are defined as clusters of cells or spots with similar SPACE from single or multiple slices.

For spatial domain detection in each slice, a distance matrix of SPACE was calculated firstly. To measure the similarities of SPACEs, SpaDo were equipped with two distance metrics, the Jensen–Shannon divergence (JSD) [50] and Manhattan distance. As a widely used measure of distribution distance, the JSD is based on the Kullback–Leibler divergence (KL) between two distributions. The KLD of SPACE between two cells or spots *P* and *Q* is defined as:

$${\text{KL}}\left(P,Q\right)=\sum {P}_{i}*{\text{log}}({P}_{i}/{Q}_{i})$$

As a symmetrized, finite, and smoothed version, JSD is defined as follows:

$${\text{JSD}}\left(P,Q\right)=({\text{KL}}\left(P,M \right)+{\text{KL}}(Q,M))/2$$

where *M* = (*P* + *Q*) / 2. A smaller JSD value indicates a higher similarity between the distributions, while a larger value suggests greater dissimilarity.

Manhattan distance (MD) is also equipped as a candidate in SpaDo software because it is much faster than JSD by sacrificing a little accuracy. MD is the sum of absolute differences between points in their cartesian coordinates and is calculated as:

$${\text{MD}}\left(P,Q\right)=\sum |{P}_{i}-{Q}_{i}|$$

Next, SpaDo detects spatial domains by applying hierarchical clustering to the distance matrix. Hierarchical clustering is performed using the hclust() function from R package with default parameters.

For multi-slice spatial domain detection, firstly, SpaDo calculates SPACE of cells or spots for each slice. Because all slices have consistent cell type annotations, i.e., they have the same embedding space, we concatenate SPACE of each slice together to calculate the JSD distance and then perform hierarchical clustering to detect spatial domain. Finally, each domain is backtracked to each slice.

### The selection of proper domain numbers

The spatial domain with different resolutions can be obtained by selecting proper domain numbers (Additional file 1: Fig. S1). To determine the optimal spatial domain number, SpaDo offers three optional strategies: (1) automatic selection using the cutreeDynamic() function with parameter “deepSplit = 2” from R package dynamicTreeCut [51]; (2) manually set by users based on their prior knowledge or specific requirements; and (3) visualization of the hierarchical trees and UMAP clustering results can assist in determining the optimal spatial domain number. The last two strategies allow for customization, providing a high level of flexibility and interpretability in the analysis of spatial domains. In this study, for each test data, different approaches were employed. If region labels were provided in the original study, the number of regions will be used as the spatial domain number. If not, SpaDo adopted the first strategy to determine the optimal spatial domain number.

### Spatial domain annotation with spatial reference

SpaDo utilizes the annotated datasets, called spatial reference, to annotate newly acquired spatial transcriptomes, referred to as spatial domain queries. Specifically, this process consists of the following four steps: (1) for each spatial domain in the spatial reference, the centroid is calculated by averaging the SPACE of all cells/spots identified as the same domain; (2) the SPACE of each cell/spot in the spatial query dataset is calculated; (3) the JSD distance between the SPACE of each cell/spot in the spatial query and each centroid of SPACE of spatial domain in the spatial reference is calculated; and (4) the spatial domain in the reference with the minimum JSD distance is assigned as the annotation for corresponding cell/spot in the spatial query.

### Multi-slice clustering analysis

Intuitively, SpaDo performs multi-slice clustering analysis by assessing the similarity between multiple slices. The similarity is calculated by spatial domain composition. Firstly, SpaDo performs the multi-slice spatial domain detection for multiple slices. Then, the spatial domain composition of individual slice is calculated, which is defined as:

$${C}^{i}=\left[\frac{{D}_{ij}}{{M}_{i}}\right],j=1,\dots N$$

where ${C}^{i}$ is a vector, meaning the spatial domain composition of the *i*-th slice, and $N$ is the number of detected domains in all slices. ${D}_{ij}$ is the number of cell/spot identified as the *j*-th domain in the *i*-th slice. If the *j*-th domain is absent in the *i*-th slice, the ${D}_{i,j}$ is set to 0. ${M}_{i}$ is the number of cell/spot in the *i-*th slice.

Finally, SpaDo performs hierarchical clustering on the spatial domain composition of all slices using pheatmap() function from R package pheatmap with default parameters.

### Parameter settings in this study

SpaDo incorporates several key parameters, including the number of k nearest neighbors for single-cell spatial transcriptomics data, searching radius for spot resolution spatial transcriptomics data, and the domain number.

In all tests conducted in this study, the number of k nearest neighbors is consistently set to 30. The selection of the domain number varies based on the test data. If region labels are available in the original study, the number of regions is used as the spatial domain number. In cases where region labels are not provided, SpaDo automatically selects the domain number using the cutreeDynamic() function with the parameter “deepSplit = 2” from the R package dynamicTreeCut [51].

Regarding the searching radius, the default value is “Radius = 2” for all test data, except for the human heart dataset [40]. For the human heart dataset [40], derived from old ST where each spot has a diameter of 100 μm and contains about 10–40 single cells [1], the searching radius is set to 1. This adjustment is made to accommodate the specific characteristics of the dataset.

### Sensitivity to distance metrics

It is important to note that SpaDo calculates the distance of SPACE for each spot or cell by default using JSD. To validate its robustness, we systematically compared SpaDo’s performance when employing various distance metrics, which include Euclidean distance, Manhattan distance, Spearman correlation, Pearson correlation, Cosine similarity, and JSD. Specifically, for Spearman, Pearson, and Cosine, where the results represent similarity within the range of − 1 to 1, the corresponding distance was obtained using “1—similarity”. This analysis provides a comprehensive evaluation of SpaDo’s stability across a spectrum of distance measurement approaches.

### Sensitivity to sequencing depth and dropouts

The sensitivity of SpaDo to sequencing depth and dropouts was assessed to account for the inherent noise in spatial transcriptomics data. Specifically, we artificially increased the dropout rate in the DLPFC_151673 and osmFISH datasets by randomly setting 10%, 30%, and 50% of the nonzero expression values to zero (Fig. 3g, h). For each dataset, *n* = 20 random dropout assignments were performed.

### The batch effects evaluation of SpaDo

SpaDo effectively addresses batch effects, as demonstrated through a comprehensive analysis involving four spot resolution DLPFC datasets (DLPFC_151673, DLPFC_151674, DLPFC_151675, DLPFC_151676), as well as three single-cell resolution MERFISH datasets. For the spot resolution DLPFC datasets, we compared the SpaDo embedding strategy SPACE with embeddings obtained from SEDR and SpaGCN, both with and without harmony [38]. Harmony was applied using default parameters. Subsequently, to evaluate the performance of SEDR and SpaGCN after incorporating harmony, we calculated the “1-Pearson correlation” as the distance between each spot embedding. In contrast to SpaDo, we refrained from using JSD in this context, given that the embeddings from SEDR and SpaGCN are not distributions and are thus unsuitable for JSD. Following this, we applied the same hierarchical clustering method as SpaDo to conduct multi-slice domain detection for SEDR and SpaGCN, with the specified domain number set at 7.

For the three MERFISH datasets, we conducted a parallel comparison involving the SpaDo embedding strategy SPACE and embeddings derived from SEDR, SpaGCN, and STAGATE, with and without harmony. We followed the same analytical steps as described above, employing default settings for domain number selection.

### Evaluation metrics

To evaluate the performance of SpaDo, ground-truth information such as the true spatial domain labels were utilized to calculated two performance metrics: adjusted rand index (ARI) and macro-F1.

For spatial domain detection with single slice, ARI was used to evaluate the performance of each method:

$${\text{ARI}}=\frac{{\sum }_{ij}\left(\begin{array}{c}{n}_{i,j}\\ 2\end{array}\right)-\left[{\sum }_{i}\left(\begin{array}{c}{a}_{i}\\ 2\end{array}\right){\sum }_{j}\left(\begin{array}{c}{b}_{j}\\ 2\end{array}\right)\right]/\left(\begin{array}{c}n\\ 2\end{array}\right)}{\frac{1}{2}\left[{\sum }_{i}\left(\begin{array}{c}{a}_{i}\\ 2\end{array}\right)+{\sum }_{j}\left(\begin{array}{c}{b}_{j}\\ 2\end{array}\right)\right]-\left[{\sum }_{i}\left(\begin{array}{c}{a}_{i}\\ 2\end{array}\right){\sum }_{j}\left(\begin{array}{c}{b}_{j}\\ 2\end{array}\right)\right]/\left(\begin{array}{c}n\\ 2\end{array}\right)}$$

where ${n}_{i,j}$ is the number of cells that are assigned to the *i*-th predicted domain label with their true domain label as the *j*-th label, ${a}_{i}={\sum }_{i}\left({n}_{ij}\right)$ and ${b}_{j}={\sum }_{j}\left({n}_{ij}\right)$.

For spatial domain annotation with multiple slices, macro-F1 was used to evaluate the performance of each method:

$${\text{macro}}-F1=\frac{1}{N}\sum_{i=1}^{N}\frac{2\times {{\text{Precision}}}_{i}\times {{\text{Recall}}}_{i}}{{{\text{Precision}}}_{i}+{{\text{Recall}}}_{i}}$$

where *N* denotes the number of spatial domains in a dataset. ${{\text{Precision}}}_{i}$ and ${{\text{Recall}}}_{i}$ are the precision and recall of the *i-*th spatial domain in the dataset.

### Benchmarking methods

In this study, we benchmarked SpaDo with Scanpy, Seurat v4, SEDR, SpaGCN, STAGATE, BayesSpace, and PASTE in different tests with default parameters (Additional file 1: Table S2).

For spatial domain detection using single-cell spatial transcriptomic data, we benchmarked Scanpy, Seurat v4, SEDR, SpaGCN, and STAGATE with default parameters. BayesSpace was excluded from this scenario as it was specifically designed for spot resolution spatial transcriptomics data. Identical number of domains was set as in the original study.

For spatial domain detection using spot level spatial transcriptomic data, we benchmarked Scanpy, Seurat v4, SEDR, SpaGCN, and BayesSpace with default parameters. STAGATE was excluded due to its occasional instability and failure in handling spot resolution data. Identical number of domains was set as in the original publications.

For reference-based spatial domain annotation, we benchmarked PASTE and Seurat v4 with default parameters.

In the sensitivity test of SpaDo combined with spot deconvolution methods, we benchmarked Cell2location against RCTD and SPOTlight. For Cell2location, the single-cell regression model was trained with default parameters and the Cell2location model was obtained with parameter detection_alpha = 20 for all datasets. Specifically, “N_cells_per_location” was set to 10 for RCC and DLPFC datasets and 20 for human and chicken heart datasets. RCTD and SPOTlight were performed with default parameters.

In all benchmarking tests, the tools were executed on a system with Intel Xeon E5-2696 v4 CPU (2.20 GHz) and GeForce GTX GPU 1080 Ti.

### Supplementary Information

**Additional file 1.** Supplementary Tables S1-S2 and Supplementary Figures S1-S14.

**Additional file 2.** Review history.

## Data Availability

The SpaDo algorithm is implemented as R package, and it is freely available under the GNU General Public License v2.0 on Github (https://github.com/bm2-lab/SpaDo) [52] and Zenodo (https://doi.org/10.5281/zenodo.10714849) [53]. All data analyzed in this paper are available in raw form from their original studies (Additional file 1: Table S1). Specifically, the osmFISH dataset [54] is available at http://linnarssonlab.org/osmFISH/osmFISH_SScortex_mouse_all_cells.loom. seqFISH + dataset [55] is available at https://github.com/CaiGroup/seqFISH-PLUS/blob/master/sourcedata.zip. The STARmap dataset [56] is available at https://www.dropbox.com/sh/f7ebheru1lbz91s/AADm6D54GSEFXB1feRy6OSASa/visual_1020/20180505_BY3_1kgenes. The MERFISH dataset [57] is available at https://datadryad.org/stash/dataset/doi:10.5061/dryad.8t8s248. The DLPFC dataset [58] is available in the spatialLIBD package (http://spatial.libd.org/spatialLIBD). For the DLPFC dataset, the corresponding single-cell reference [59] used for spot deconvolution is available at https://libd-snrnaseq-pilot.s3.us-east-2.amazonaws.com/SCE_DLPFC-n3_tran-etal.rda. The RCC dataset is from GSE175540 [60]. For RCC dataset, the corresponding single-cell reference [61] is available at https://singlecell.broadinstitute.org/single_cell/study/SCP1288/tumor-and-immune-reprogramming-during-immunotherapy-in-advanced-renal-cell-carcinoma#study-download. For all the above datasets, domain labels are from their original studies. The human heart dataset [62] is available at https://data.mendeley.com/datasets/mbvhhf8m62/2/files/f76ec6ad-addd-41c3-9eec-56e31ddbac71. For human heart dataset, the corresponding single-cell reference [63] we used for spot deconvolution is available at https://data.mendeley.com/public-files/datasets/mbvhhf8m62/files/33fb42ae-7b40-4a70-b61d-676f44d68d4c/file_downloaded. The chicken heart dataset and the corresponding single-cell reference for spot deconvolution is from GSE149457 [64]. The organoid dataset [65] is available at https://singlecell.broadinstitute.org/single_cell/study/SCP1756/cortical-organoids-atlas.
