# Supplementary material for: Multi-slice spatial transcriptome domain analysis with SpaDo
Source: Genome Biol. 2024 Mar 19;25:73. doi: 10.1186/s13059-024-03213-x (PMC10949687; doi:10.1186/s13059-024-03213-x)
Supplement: Supplementary file 1 — Additional file 1. Supplementary Tables S1-S2 and Supplementary Figures S1-S14. [file 13059_2024_3213_MOESM1_ESM.pdf]

Additional file 1 for "Multi-slice Spatial Transcriptome Domain Analysis with SpaDo".

This file includes:

Tables S1 to S2

Figures S1 to S14

Table S1: Details of data tested in this paper.

Tissue	Technology	Resolution (Spot/Single cell)	No. of slice	Accession
Mouse visual cortex[10]	STARmap	Single cell	1	https://www.dropbox.com/sh/f7ebheru1lbz91s/AADm6D54GSEFXB1feRy6OSASa/visual_1020/20180505_BY3_1kgenes
Cortex[8]	seqFISH+	Single cell	1	https://github.com/CaiGroup/seqFISH-PLUS/blob/master/sourcedata.zip
Somatosensory cortex[9]	osmFISH	Single cell	1	http://linnarssonlab.org/osmFISH/osmFISH_SScortex_mouse_all_cells.loom
Hypothalamic preoptic region[46]	MERFISH	Single cell	3	https://datadryad.org/stash/dataset/doi:10.5061/dryad.8t8s248
Dorsolateral prefrontal cortex (DLPFC) [31]	10x Visium	Spot	12	Accessible within the spatialLIBD package(http://spatial.libd.org/spatialLIBD)
Renal cell carcinoma (RCC) [32]	10x Visium	Spot	5	GSE175540
Human heart of development[38]	Old ST	Spot	19	https://data.mendeley.com/datasets/mbvhhf8m62/2/files/f76ec6ad-addd-41c3-9eec-56e31ddbac71
Human cortical organoid of development[39]	Slide-seq V2	Spot	9	https://singlecell.broadinstitute.org/single_cell/study/SCP1756/cortical-organoids-atlas
Chicken heart of development	10x Visium	Spot	11	GSE149457

Table S2. The benchmarked methods in different scenarios.

Tools	Algrithom	Supported resolution	Tested scenarios
Scanpy[15]	Leiden clustering	Single-cell and spot-resolution	● Domain detection in single-cell spatial transcriptomic data.<br>● Domain detection in spot-resolution spatial transcriptomic data.
Seurat[29]	Louvain clustering	Single-cell and spot-resolution	● Domain detection in single-cell spatial transcriptomic data<br>● Domain detection in spot-resolution spatial transcriptomic data.<br>● Spatial domain annotation.
SEDR[18]	GNN (Graph Neural Network)	Single-cell and spot-resolution	● Domain detection in single-cell spatial transcriptomic data.<br>● Domain detection in spot-resolution spatial transcriptomic data.
STAGATE[19]	GNN	Single-cell and spot resolution	● Domain detection in single-cell spatial transcriptomic data.
SpaGCN[17]	GNN	Single-cell and spot resolution	● Domain detection in single-cell spatial transcriptomic data.<br>● Domain detection in spot-resolution spatial transcriptomic data.
BayeSpace[16]	Bayesian approach	Spot-resolution	● Domain detection in spot-resolution spatial transcriptomic data.
PASTE[22]	Optimal transport model	Single-cell and spot-resolution	● Spatial domain annotation.

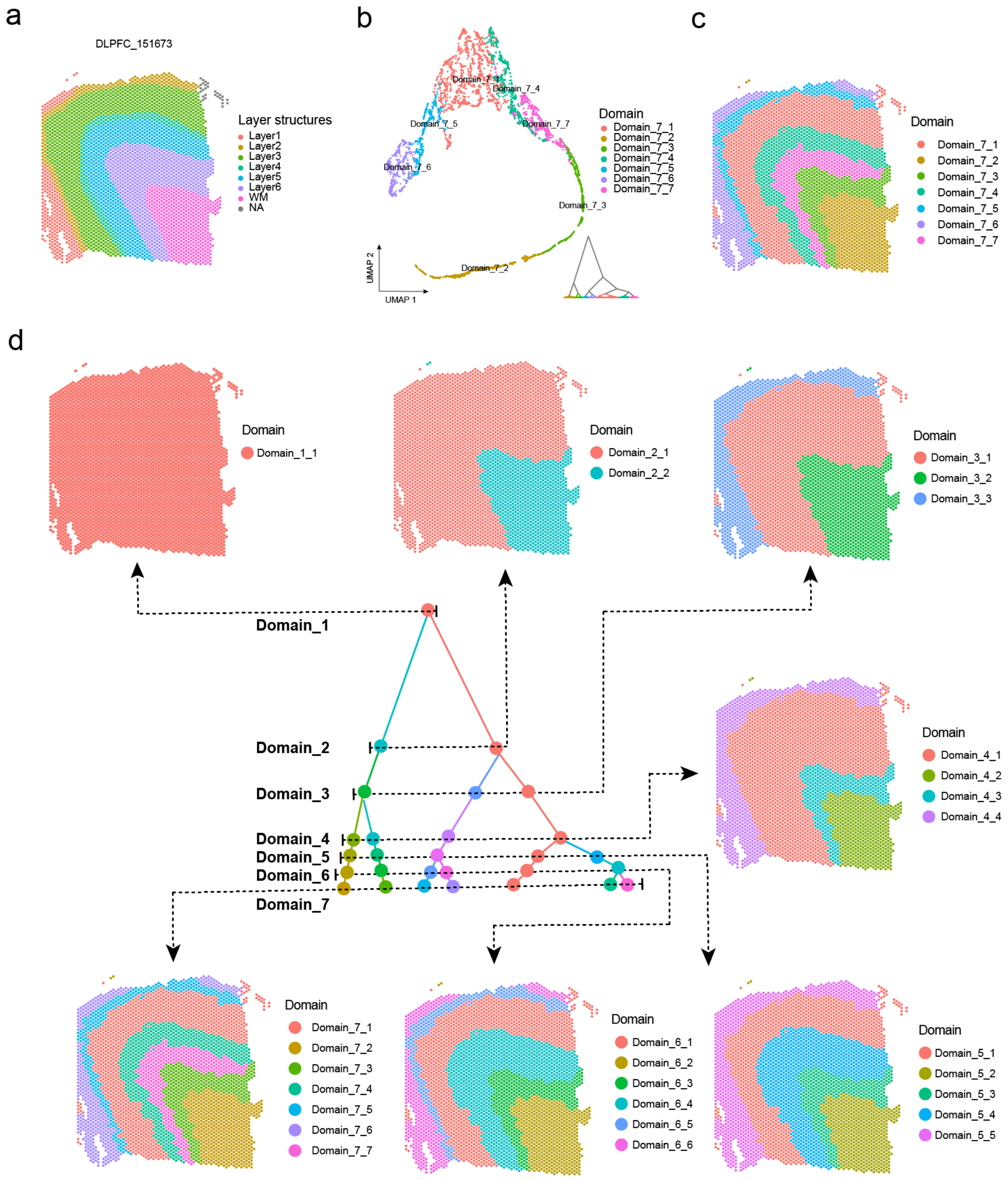

Fig. S1 SpaDo detectes spatial domain with a multi-resolution schema hieratically. **a** Manually annotated layer structure of DLPFC_151673 dataset. **b** The UMAP visualization of SPACE and the corresponding hierarchical clustering result. **c** Location of spatial domains detected by SpaDo. **d** Location of the multi-resolution spatial domains detected by SpaDo.

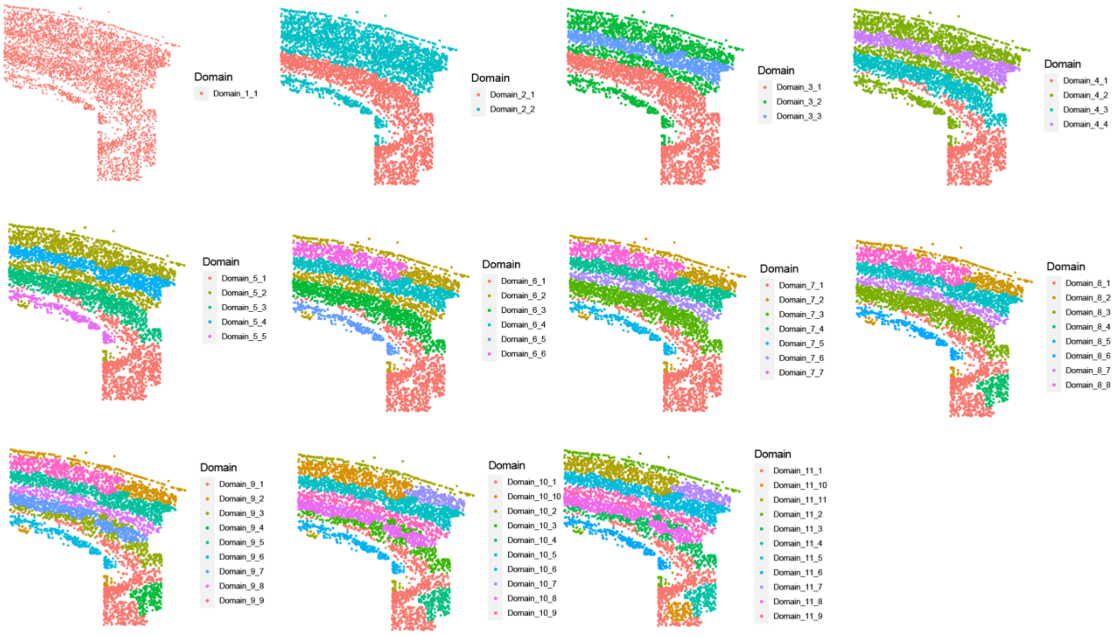

Fig. S2 The multi-resolution spatial domain detection results of osmFISH data.

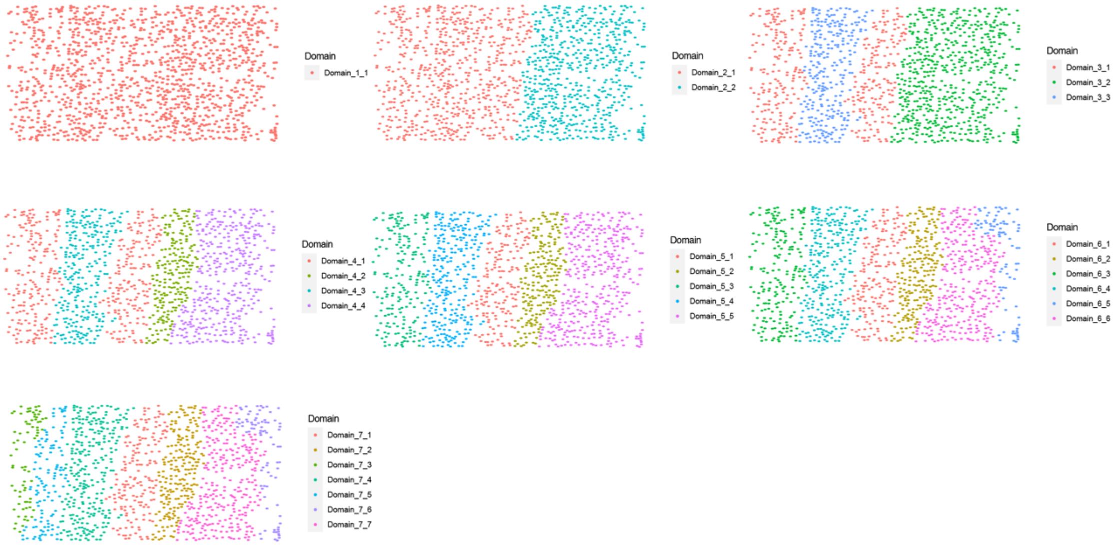

Fig. S3 The multi-resolution spatial domain detection results of STARmap data.

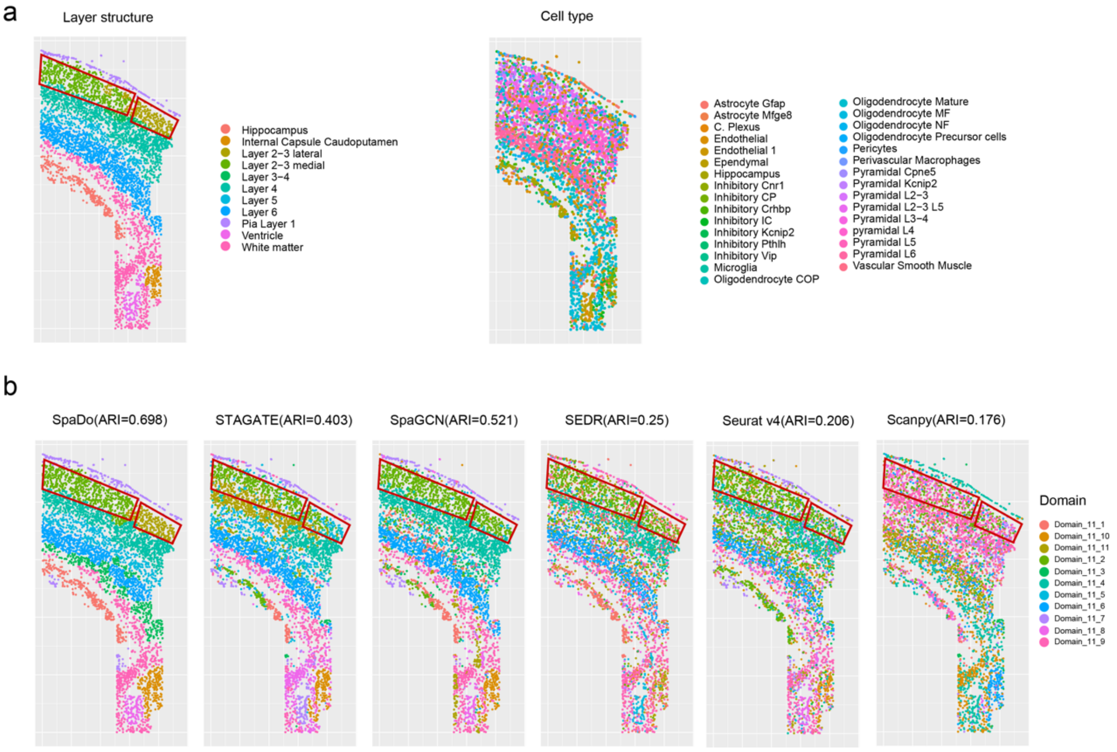

Fig. S4. Details of benchmark in osmFISH data related to Fig. 2a. **a** Layer structure and cell type of osmFISH data from the original paper. **b** ARI and visualization of domain detection for each method. The regions in the red rectangular are examples showing the difference of detected spatial domains by SpaDo and other methods.

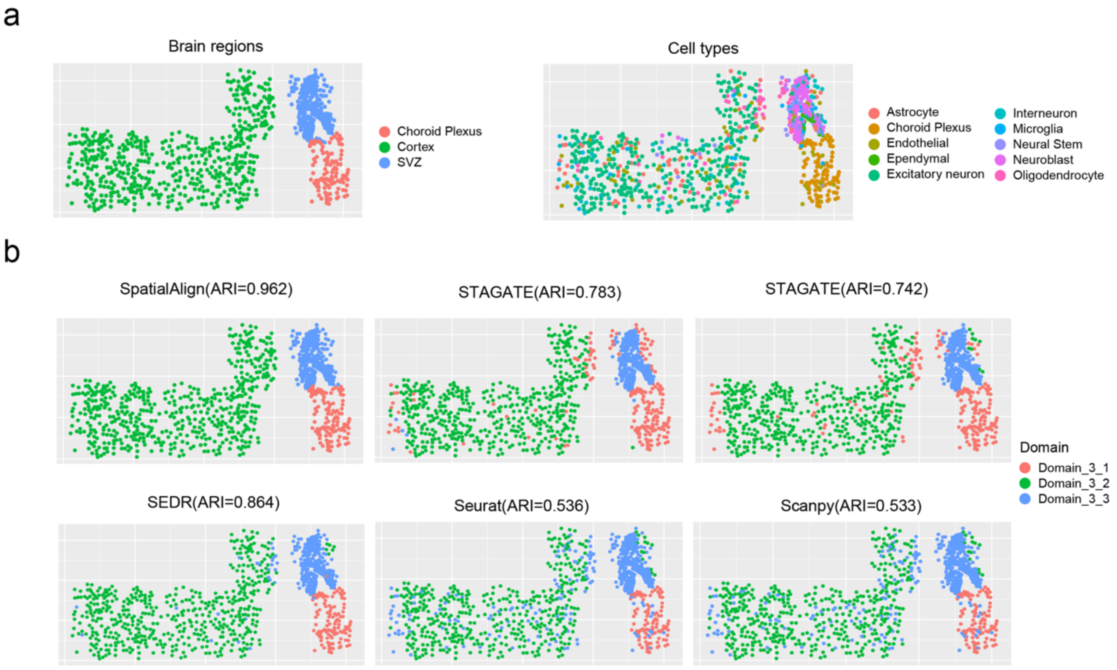

Fig. S5 Details of benchmark in seqFISH+ data related to Fig. 2a. **a** Brain regions and cell types of seqFISH+ data from the original paper. **b** ARI and visualization of domain detection for each method.

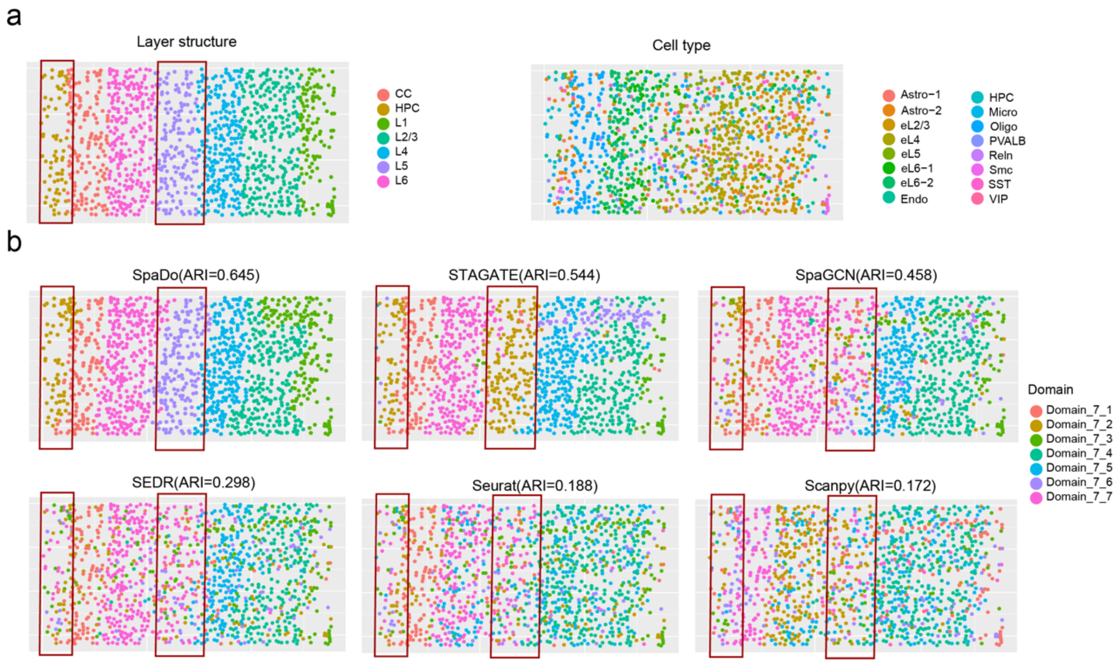

Fig. S6 Details of benchmark in STARmap data related to Fig. 2a. **a** Brain regions and cell types of STARmap data from the original paper. **b** ARI and visualization of domain detection for each method. The regions in the red rectangular are examples showing the difference of detected spatial domains by SpaDo and other methods.

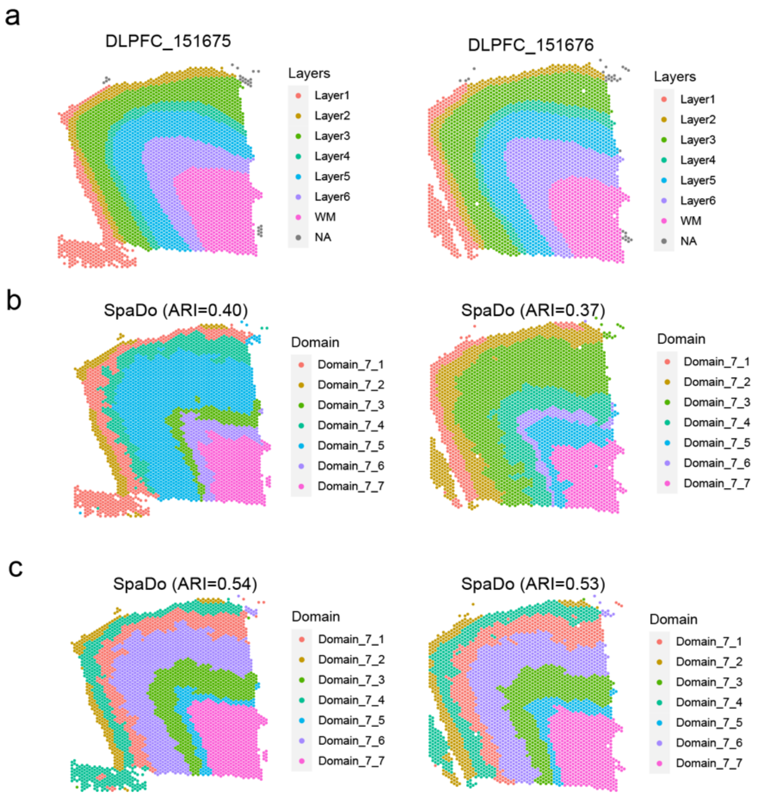

Fig. S7 Performance of SpaDo can be further improved by integrating multiple slices. **a** Manually annotated labels of layers for DLPFC_151675 and DLPFC_151676 datasets. **b** Location and ARI of SpaDo for single slice. **c** Location and ARI of SpaDo by integrating DLPFC_151675 and DLPFC_151676 dataset.

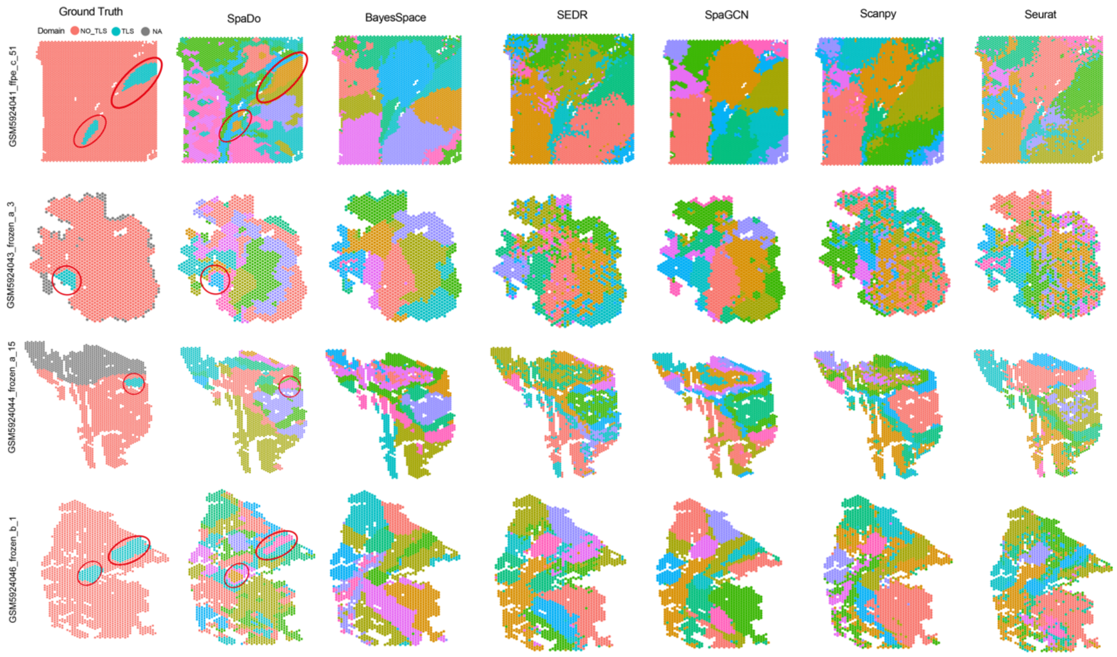

Fig. S8 Benchmarking TLS-like domain detection when domain number is set to 10 related to Fig. 2e.

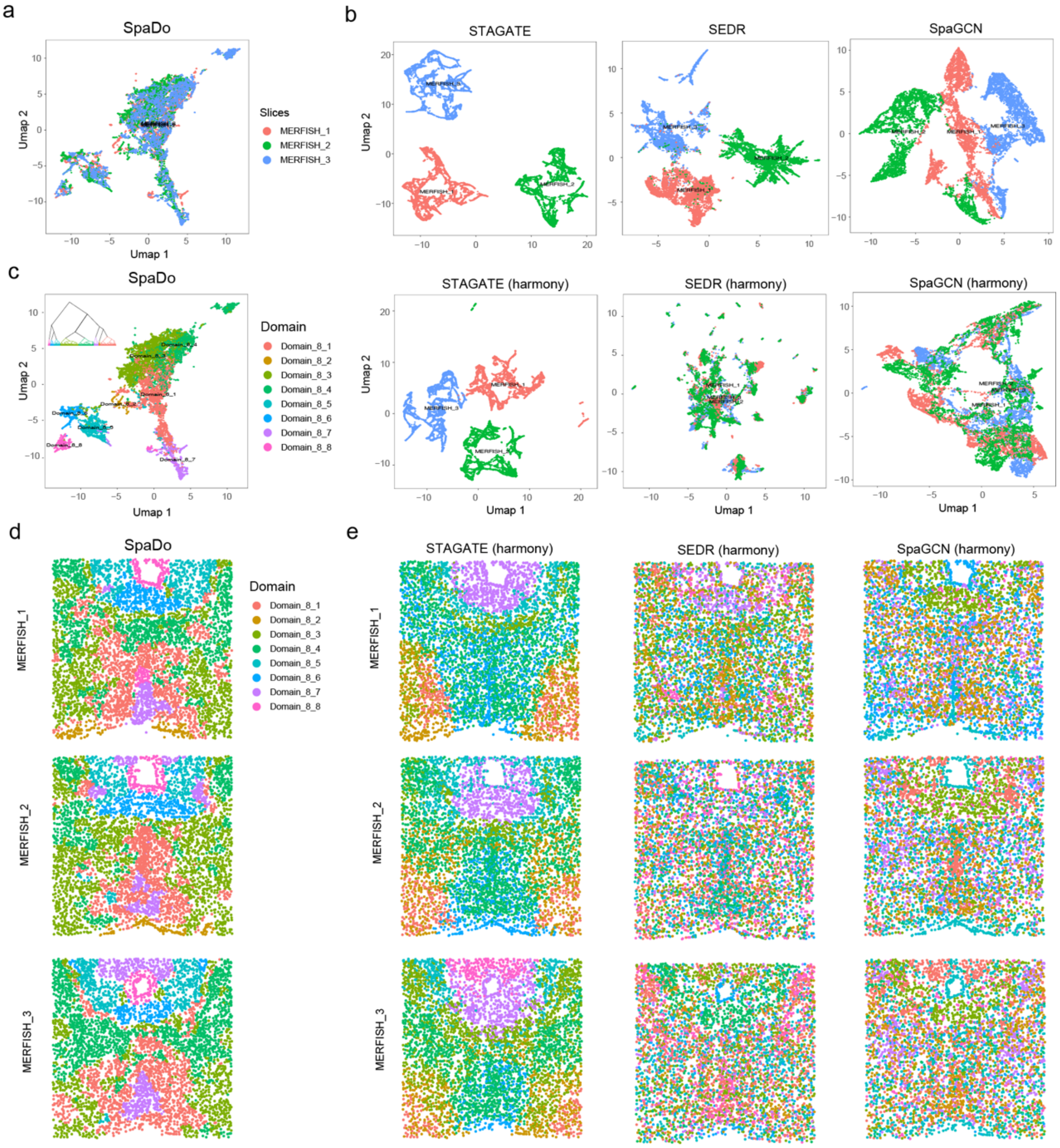

Fig. S9. The batch effects evaluation of SpaDo and other existing methods for multi-slice domain detection on MERFISH data. **a** Umap of SpaDo on 3 MERFISH slices (Colored by slices). **b** Umap of SEDR and SpaGCN with and without harmony on 3 MERFISH slices (Colored by slices). **c** Umap and hierarchical clustering result of SpaDo on 3 MERFISH slices (Colored by detected spatial domains). **d-f** Locations of spatial domains annotated by SpaDo, SEDR with harmony, SpaGCN with harmony on 3 MERFISH slices.

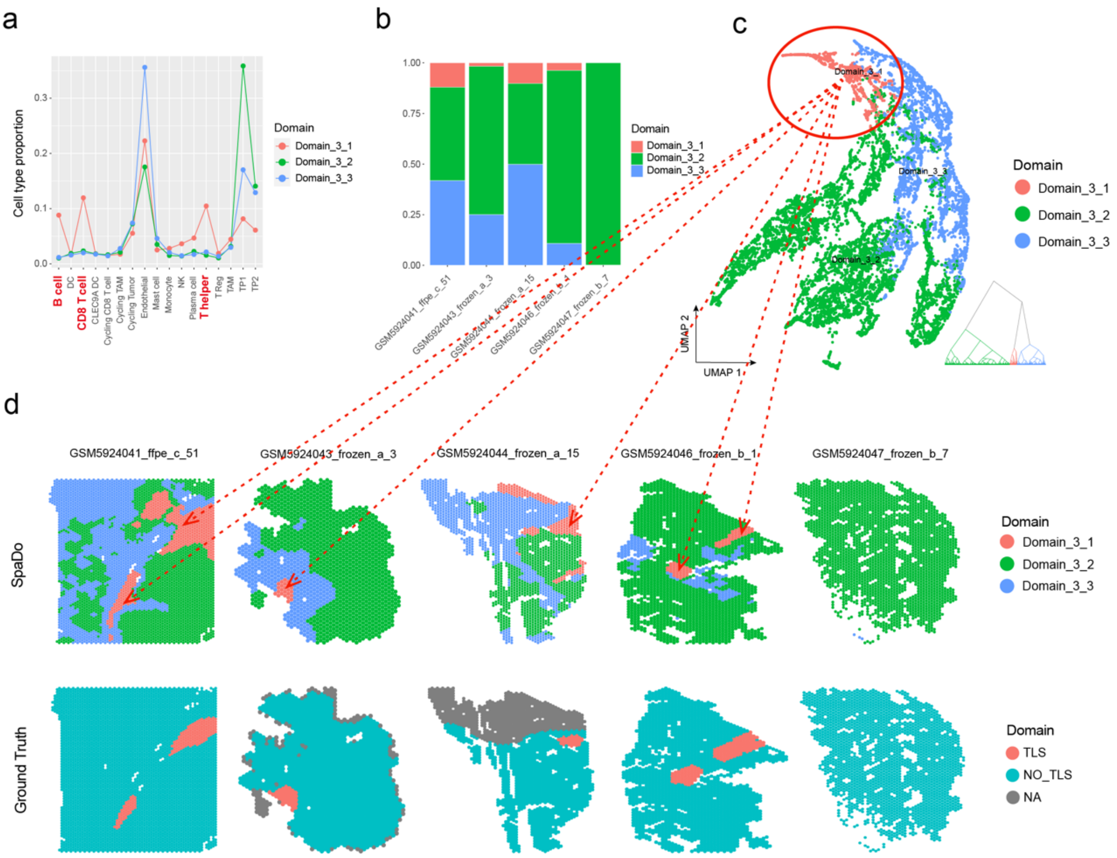

Fig. S10 SpaDo enables cross-slice spatial domain detection when spatial domain number is set to 3. **a** Cell type proportion of each detected spatial domain. **b** Proportion of detected spatial domains for each RCC slice. **c** Umap and hierarchical clustering result of SPACE on 5 RCC slices. **d** Location of detected spatial domains against true TLS labels in all 5 RCC slices.

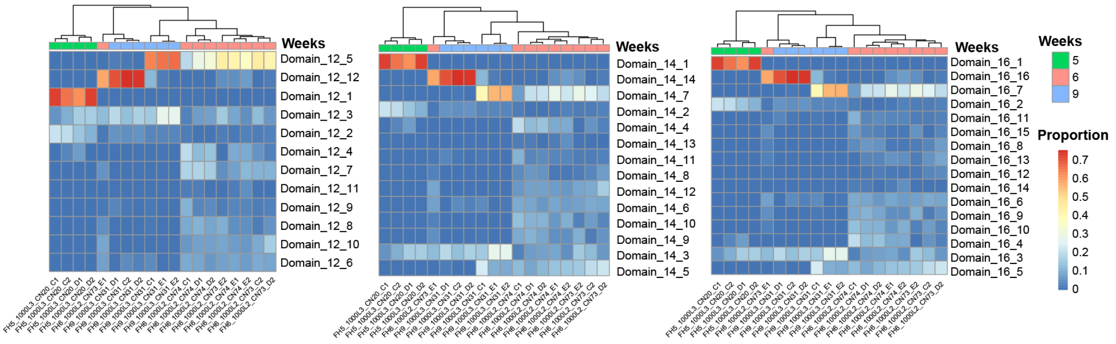

Fig. S11 The clustering results of SpaDo with different domain number in human heart dataset related to Fig. 7f.

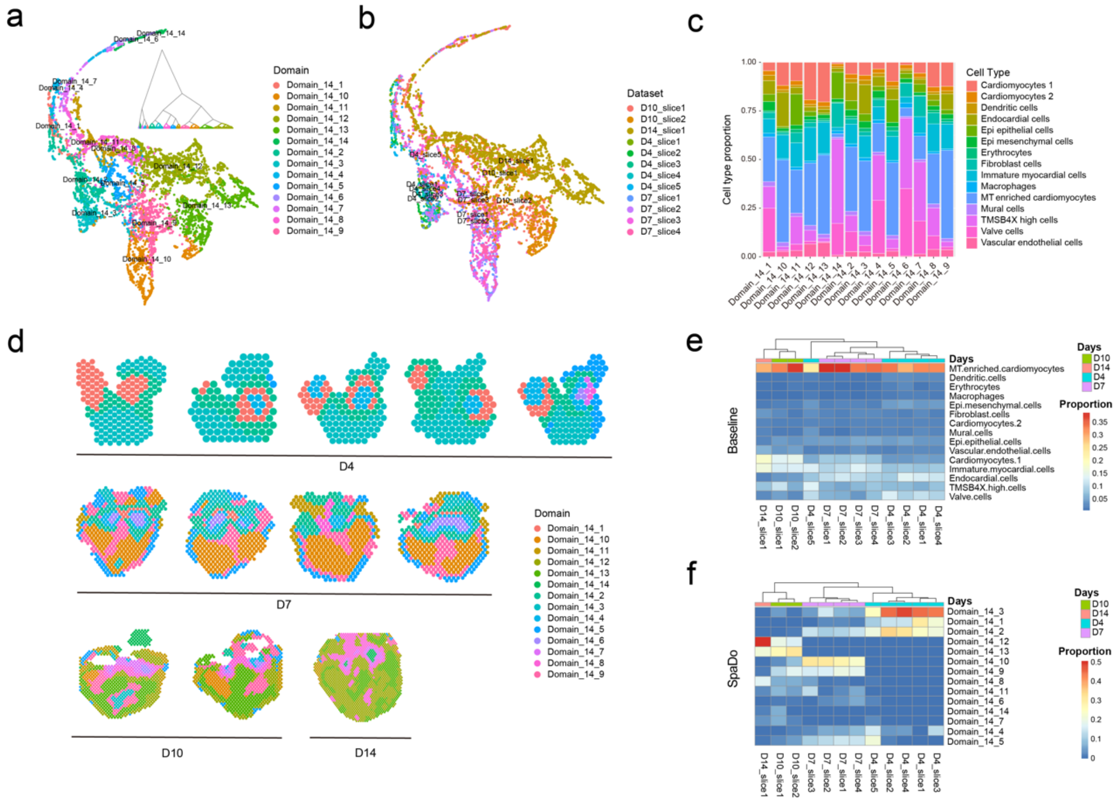

Fig. S12 SpaDo enables slice-level clustering analysis in chicken heart dataset. **a** Umap and hierarchical clustering result of SPACE on 12 chicken heart slices. (Colored by detected spatial domains). **b** Umap of SPACE on 12 chicken heart slices. (Colored by slices). **c** Cell type proportion of each detected domain. **d** Location of each detected domain in 12 slices. **e** Heatmap of clustering results of 12 slices using average cell type proportion as baseline. **f** Heatmap of clustering results of 12 slices using domain composition detected by SpaDo.

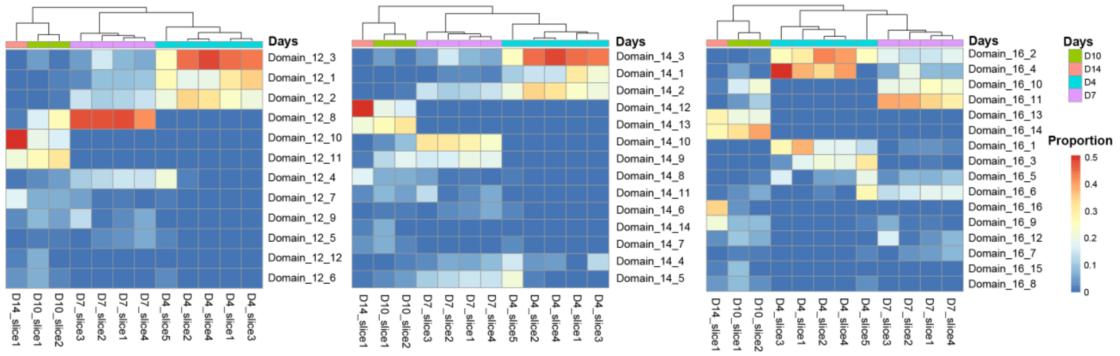

Fig. S13 The clustering results of SpaDo with different domain number in chicken heart dataset related to Fig. S12.

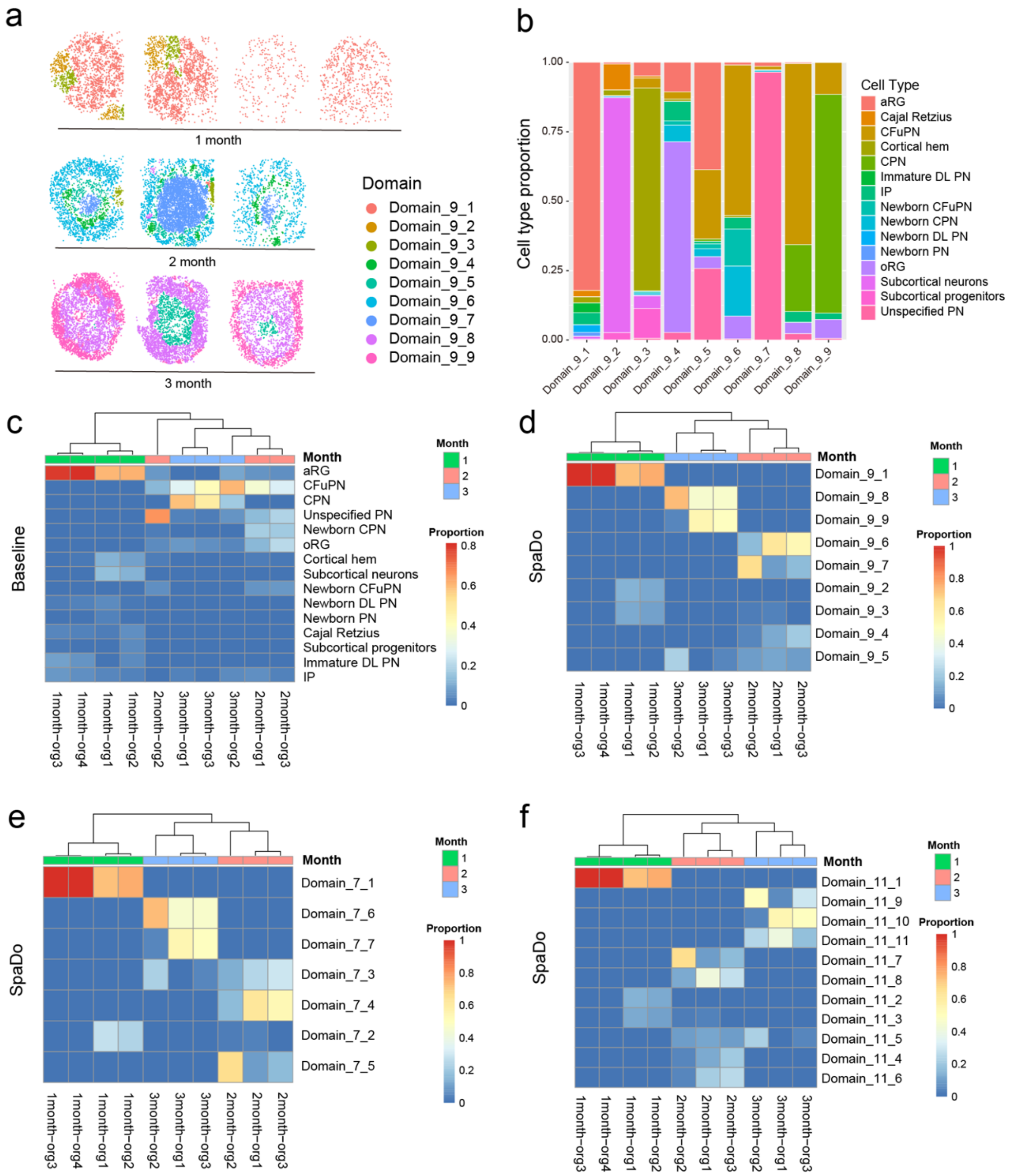

Fig. S14 SpaDo enables slice-level clustering analysis in human cortical organoid dataset. **a** Location of each detected domain on 10 cortical organoid slices. **b** Cell type proportion of each detected domain. **c** Heatmap of clustering results of 10 cortical organoid slices using average cell type proportion as baseline. **d** Heatmap of clustering results of 10 cortical organoid slices using domain composition detected by SpaDo. **e** Clustering results of SpaDo with different domain number (domain number is set to 7). **f** Clustering results of SpaDo with different domain number (domain number is set to 11).
