# Supplementary material for: Multi-slice spatial transcriptome domain analysis with SpaDo
Source: Genome Biol. 2024 Mar 19;25:73. doi: 10.1186/s13059-024-03213-x (PMC10949687; doi:10.1186/s13059-024-03213-x)
Supplement: Supplementary file 2 — Additional file 2. Review history. [file 13059_2024_3213_MOESM2_ESM.docx]

**1^st^ round**

**Reviewer 1**

The authors present SpaDo, an integrative tool focused on the analysis of "spatial domain", including spatial domain detection for single and multiple slices, spatial domain annotation and cross-slice clustering at single-cell and spot resolutions. SpaDo designed a simple yet effective embedding strategy, spatially adjacent cell type embedding (SPACE) to integrate gene expression and spatial information, which is novel and reasonable. The authors conducted comprehensive analysis to demonstrate the superiority of SpaDo, including benchmarking with other existing approaches and providing many robustness tests and illustating multiple applications of SpaDo in different scenarios. SpaDo was proved to be fast and with low memory usage. In addition, the authors provided detailed user tutorial and different demo datasets in the Github site. Spatial transcriptome data is noisy and heterogenous, thus robustness test of the method is very important, and the authors conducted lots of robustness tests, which is appreciated. Generally, SpaDo is novel and seems reliable, which can be potentially useful in spatial transcriptome data analysis. However, there are a few concerns that should be addressed before this manuscript can be accepted for publication.

Major comments:
1. The authors first proved the superiority of SpaDo in the task of single slice spatial domain detection and they showed the robustness of parameter "Radius" (Fig. 3d). Why not compare "Radius=1" and what happens when "Radius=1"?
2. Jensen Shannon Divergence (JSD) was used to calculate the distance of SPACE of different cells, which is reasonable but not proved. I wonder if SpaDo robust to other similarity metrics, such as Pearson, Spearman or Cosine?
3. Batch effects are often an important issue when integrating multiple sources data. I wonder if there are batch effects of SPACE within different slices?
4. Spatial omics datasets are increasingly accumulating, and I hope the authors could disscuss more about the possibility of SpaDo in the multi-modality spatial data analysis.
5. Resolving the spatial heterogeneity of tumors is very important to explore the pathogenesis as well as the treatment of tumors, and I hope that the authors can discuss whether SpaDo can be used to infer the common tumor evolution models by integrating multiple spatial slices.
6. SpaDo could detect the common and unique spatial domains among different slices, then whether these spatial domains can be potential spatial markers?
7. SpaDo can measure the overall similarity of different slices, which provides richer information for classifying samples, so I wonder if SpaDo could be used to classify different tumour patients in order to provide precise treatment from the perspective of spatial heterogeneity?
8. A detailed description of Jensen Shannon Divergence may be necessary in the methods part.
9. In the Github site, the authors provided detailed tutorials and lots of demo datasets, which is appreciated. However, in the line 58 of "SpaDo_Tutorial_ok.html", there was an "ERROR" message, which should be fixed.

Minor comments:
1. How to select the optimal spatial domain number is important, and it will be clearer to have this part as a separate section in the methods part.
2. It is necessary to state how many spots in dataset "DLPFC_151673" when comparing runtime and memory usage.
3. Currently there are several versions of Seurat, and authors need to specify which version they are using.
4. In Fig. 5d-g, I think it will be clearer to provide the macro-F1 of each slice.
5. In Fig. 4a, b and Fig 6c, "Proportion" should be replaced with "Cell type proportion" and in Fig. 4c, "Proportion" should be replaced with "Domain proportion", which will be clearer.

**Reviewer 2**

This study presents a novel approach for the analysis of spatial transcriptomics data across multiple datasets.

Approach:
In this work, a multi-step approach for analysis was devised. It can be characterized as a region-smoothing technique utilizing cell type compositions. While this design can effectively address the batch issue across layers, I have several concerns to raise:

The performance heavily relies on the identification of cell types or cell compositions from external methods.
The regional composition (SPACE) can capture diversity to some extent, but it shares similarities with existing spatial embedding methods, such as many spatial graph approaches in the field. I'm hesitant to conclude that this embedding surpasses the efficacy of graph neural network approaches that utilize high-dimensional gene expressions as input, as opposed to only cell type percentages (which may represent just a few dimensions). I am particularly concerned that this approach may oversmooth features and diminish tissue heterogeneity.
I don't perceive this approach as a multi-slice technique. It concatenates representations from multiple slices (simply combining them along the sample dimension). It may be worthwhile to explore features that directly address the multi-slice issue, rather than relying on a concatenation approach. Any representation approaches can handle mutli-slice issue in this way.
Clarification is needed on how hierarchical clustering cutoffs are determined.

Results:
In the introduction:
"However, these methods are restricted with high computational complexity, limited interpretability, and specific and strict assumptions." This statement appears unfair. Some of these methods are straightforward, while others have a solid statistical foundation.
"Although existing methods can detect spatial domains within a single tissue slice, they cannot directly handle cross-slice spatial domain analysis for multiple tissue slices in general." With the application of batch removal, concatenation, and clustering, most existing approaches can indeed be adapted for multi-slice tasks.
Figure 2: The visualization of hierarchical clustering operation lacks significance here, as it is a standard approach.
Supplementary Figure 4: More detailed annotations, including those pertaining to glutamatergic neural layers (as shown in original paper), could enhance the utility of this figure.

Overall, the authors conducted experiments on numerous datasets; however, I find that the proposed approach did not address the multi-slice issue and may not introduce sufficient innovation for Genome Biology.

**Reviewer 3**

The authors proposed a spatial transcriptomics domain detection method, SpaDo, that enables domain analysis across multiple slices. The authors demonstrated that through integrating multiple slices, SpaDo more accurately detected spatial domains comparing to existing methods, and is able to detect consensus domains across slices, perform domain annotation, and slice clustering. The paper is well written and extensive experiments were performed. However, I still have some concerns, especially regarding the evaluation and understanding how SpaDo achieved improved domain detection accuracy:

1. SpaDo detects domains at multiple levels of resolution. It's unclear which level of resolution was used for the evaluation in Fig.3. In addition, the other methods, e.g., Seurat, Scanpy, SpaGCN can also detect domains at different resolution by tuning the clustering resolution parameters. It's questionable whether the other methods achieved their best performance, as it seems that the authors didn't tune the resolution parameters for those methods.
2. SpaDo uses cell2location as the starting point for spot resolution data. However, different cell type deconvolution methods usually generate results that are inconsistent with each other. It's important to know how sensitive is SpaDo to different cell type deconvolution methods. Is cell2location always the best option for different datasets?
3. There are usually batch effects for different slices, especially for those sampled from different individuals. However, the authors didn't discuss the batch effects in different slices and there is not a clear procedure of removing batch effects. The authors should discuss how the batch effect was handled in SpaDo and how could it possibly influence the performance of SpaDo.
4. The most important contribution of SpaDo is its improved accuracy in detecting spatial domains by integrating multiple slices. However, it is unclear that how much the multiple slices contribute to the accuracy comparing to using single slice. The authors should perform ablation studies that use SpaDo on each single slice and compare with the results from combining multiple slices.
5. When using Seurat clustering as the cell type annotation, SpaDo is basically a spatial smoothing of the Seurat cell clusters. However, in Fig. 3e, SpaDo detects domains where no clustering signal could be observed from the Seurat domains. The authors should discuss why SpaDo significantly outperformed Seurat when using it as the starting point.

**Authors’ response**

Reviewer #1:

Thank you for your valuable insights. We have thoroughly addressed your significant comments, specifically tackling issues related to the comparison of "Radius=1" in SpaDo, the tolerance to diverse similarity metrics, batch effects across distinct slices, and the potential applicability of SpaDo in multi-modality spatial data analysis and tumor evolution modeling. The revised manuscript expanded discussions on these topics.

Furthermore, we have addressed the minor comments, including the optimal selection of spatial domain numbers, the clarification of the number of spots in a dataset, the clarification of Seurat version usage, and the elucidation of terms in figures.

Below are point-to-point responses to each of your comments:

Major comments:

1. The authors first proved the superiority of SpaDo in the task of single slice spatial domain detection and they showed the robustness of parameter "Radius" (Fig. 3d). Why not compare "Radius=1" and what happens when "Radius=1"?

Response:

Thank you for your comment. We did not initially compare "Radius=1" in our SpaDo study as, SpaDo might not effectively utilize spatial information at this setting. However, in response to your suggestion, we have now included this comparison in Fig. 3b of the revised manuscript. The updated figure clearly demonstrates that the performance of "Radius=1" is significantly lower than other radius values, serving as a baseline. Further details and clarifications are shown below and have been added to pages 7-8 of the revised manuscript.

“It's worth noting that when Radius=1, SpaDo may not fully leverage spatial information unless the spot radius for spatial transcriptome sequencing methods is very large, such as old ST, so it is lower than other radius value significantly, which is included here solely as a baseline.”

2. Jensen Shannon Divergence (JSD) was used to calculate the distance of SPACE of different cells, which is reasonable but not proved. I wonder if SpaDo robust to other similarity metrics, such as Pearson, Spearman or Cosine?

Response:

Thank you for your valuable comment. We now expanded evaluation of similarity metrics in the revised manuscript. We assessed SpaDo's performance using various commonly employed metrics, including Spearman correlation, Pearson coefficient, Cosine distance, Euclidean distance, Manhattan distance, and JSD, for both single-cell (Fig. 3e) and spot resolution spatial transcriptomics data (Fig. 3f). Our findings consistently demonstrate the robustness of SpaDo across these diverse distance metrics, with JSD consistently yielding the highest median performance. We appreciate your suggestion, and this additional analysis enhances the comprehensiveness of our study. Further details and clarifications have been added to page 7-8 and methods section titled “Sensitivity to distance metrics” (Page 20) of the revised manuscript.

3. Batch effects are often an important issue when integrating multiple sources data. I wonder if there are batch effects of SPACE within different slices?

Response:

Thank you for your valuable comment. In our revised manuscript, we conducted a comprehensive comparison between SpaDo and other existing methods for addressing batch effects in both single-cell and spot resolution spatial transcriptomics data (Fig. 4 and Additional File 1: Fig. S9).

Specifically, for spot resolution spatial transcriptomics, we tested 4 DLPFC slices belong to the same sample, each with the same seven layers. We compared the performance of SPACE utilized in SpaDo (Fig. 4a) with the embeddings obtained by SEDR and SpaGCN, both with and without harmony to correct batch effects (Fig. 4c). The results clearly demonstrate the effectiveness of SpaDo in removing batch effects, whereas SEDR and SpaGCN exhibit significant batch effects even when combined with harmony.

Furthermore, we conducted multi-slice domain detection with SpaDo (Fig. 4c, d). For embeddings obtained by combining harmony with SEDR and SpaGCN, we employed a similar strategy for multi-slice domain detection (Fig. 4e, f). The results highlight that only SpaDo consistently obtains domain results across the four DLPFC slices (Fig. 4d-f).
Similar conclusions were drawn from our analysis of three single cell MERFISH
datasets (Additional File 1: Fig. S9). We appreciate your attention to this crucial aspect,
and these findings underscore SpaDo's efficacy in addressing batch effects across
different slices. Further details and clarifications have been added to pages 8-10 and
methods section titled “The batch effects evaluation of SpaDo” (Page 20-21) of the
revised manuscript.

4. Spatial omics datasets are increasingly accumulating, and I hope the authors could
disscuss more about the possibility of SpaDo in the multi-modality spatial data
analysis.
Response:
Thank you for your suggestion. We now expanded the discussion on the potential of
SpaDo in the analysis of multi-modality spatial data. The relevant details are shown
below and can be found on page 15-16 of the revised manuscript.
“While our study primarily focuses on applying SpaDo to spatial transcriptomic data, it's
noteworthy that SpaDo holds the potential for extension into multimodality spatial data
analysis. This extension could be particularly valuable if corresponding cell types
across different omics datasets are identifiable. Notably, advancements in spatial
epigenomics[42, 43] and technologies like slide-DNA-seq[44] present exciting
opportunities for integrating epigenetic and DNA information into spatial analyses.”

5. Resolving the spatial heterogeneity of tumors is very important to explore the
pathogenesis as well as the treatment of tumors, and I hope that the authors can
discuss whether SpaDo can be used to infer the common tumor evolution models by
integrating multiple spatial slices.
Response:
Thank you for this valuable comment. We now expanded our discussion on the
potential of SpaDo to infer common tumor evolution models by integrating multiple
spatial slices. With the ongoing evolution of spatial DNA-seq technologies, such as
slide-DNA-seq, there is a growing potential to decipher more accurate tumor evolution
patterns. SpaDo is well-positioned to capitalize on these advancements, allowing the
integration of spatial multimodal data from multiple slices. The relevant details are
shown below and can be found on page 15-16 of the revised manuscript.
“As spatial DNA-seq technologies like slide-DNA-seq continue to evolve, they offer the
potential to decipher more accurate tumor evolution patterns[45]. SpaDo, in turn, is
poised to leverage these developments and combine spatial multimodal data from
multiple slices.”

6. SpaDo could detect the common and unique spatial domains among different slices,
then whether these spatial domains can be potential spatial markers?
Response:
Thank you for your valuable insight. Indeed, the spatial domains detected by SpaDo
among different slices have the potential to serve as spatial markers. We have
incorporated this acknowledgment and provided further elaboration in the revised
manuscript's discussion (shown below), specifically on page 16.
“This integration holds the promise of uncovering new tumor markers, including both
consistent and differential tumor evolution patterns, thereby contributing to a deeper
understanding of spatial biology.”

7. SpaDo can measure the overall similarity of different slices, which provides richer
information for classifying samples, so I wonder if SpaDo could be used to classify
different tumour patients in order to provide precise treatment from the perspective of
spatial heterogeneity?
Response:
Thank you for your valuable comment. While SpaDo can effectively measure the
overall similarity of different slices, it is challenging to use this information for
classifying tumor patients accurately. The wide variation in sampling sites contributes
to difficulties in representing the true similarity between patients. Instead, we find that
comparing key spatial domains, such as TLS, is more suitable for measuring the
similarity between different tumor patients.

8. A detailed description of Jensen Shannon Divergence may be necessary in the methods part.

Response:

Thank you for your valuable suggestion. We have addressed it by incorporating a detailed description of Jensen Shannon Divergence in the methods section. You can find this information on page 18 of the revised manuscript.

9. In the Github site, the authors provided detailed tutorials and lots of demo datasets, which is appreciated. However, in the line 58 of "SpaDo_Tutorial_ok.html", there was an "ERROR" message, which should be fixed.

Response:

Thanks for this valuable comments. We have addressed the issue, and the error message in line 58 of "SpaDo_Tutorial_ok.html" has been corrected. We appreciate your diligence in reviewing the tutorial and demo datasets on our GitHub site.

Minor comments:

1. How to select the optimal spatial domain number is important, and it will be clearer to have this part as a separate section in the methods part.

Response:

Thank you for your valuable suggestion. We have added a separate section titled "The selection of proper domain numbers" in pages 19 of the methods part in our revised manuscript. This new section explains the process of selecting the optimal spatial domain number.

2. It is necessary to state how many spots in dataset "DLPFC_151673" when comparing runtime and memory usage.

Response:

Thank you for your valuable comment. We have included the information about the number of spots in the dataset "DLPFC_151673" when comparing runtime and memory usage. This addition can be found in the figure caption of Fig. 2 on page 6 in the revised manuscript.

3. Currently there are several versions of Seurat, and authors need to specify which version they are using.

Response:

Thank you for your comment. In the revised manuscript, we specified that we used Seurat v4 instead of the earlier versions.

4. In Fig. 5d-g, I think it will be clearer to provide the macro-F1 of each slice.

Response:

Thank you for your suggestion. We have included the macro-F1 values for each slice in Fig. 6 of the revised manuscript.

5. In Fig. 4a, b and Fig 6c, "Proportion" should be replaced with "Cell type proportion" and in Fig. 4c, "Proportion" should be replaced with "Domain proportion", which will be clearer.

Response:

Thank you for your valuable feedback. We have made the necessary revisions in our revised manuscript.

Reviewer #2:

Thank you for your detailed review. We have addressed concerns about SpaDo's reliance on external methods for cell type identification, clarified the superiority of the SPACE embedding, provided explanations for multi-slice integration, and added details on hierarchical clustering cutoffs. We also revised the introduction for fairness, compared SpaDo with existing methods, adjusted visualizations, and explained the intentional focus in Supplementary Figure 4.

We believe these revisions enhance the manuscript's clarity and we appreciate your valuable feedback.

Below are point-to-point responses to each of your comments:

1.The performance heavily relies on the identification of cell types or cell compositions from external methods.

Response:

Thank you for your valuable comment. Indeed, SpaDo relies on the identification of cell types or cell compositions from external methods, such as Seurat or Cell2location. In response to this concern, we have conducted a comprehensive robust test, as shown in Figure 3c and d, to evaluate SpaDo's performance. The results of the test indicate that SpaDo exhibits robustness across different cell type or cell composition identification methods.

As a result, SpaDo indeed heavily depends on cell type annotation methods like Seurat v4 and Cell2location. However, SpaDo has demonstrated robustness to these methods (Fig. 3c, d). Moreover, advancements in more accurate cell type annotation and spot deconvolution methods could potentially enhance SpaDo's performance. We added these descriptions in the discussion of our revised manuscript in page 15 (shown below).

“SpaDo have two potential limitations. Firstly, it heavily depends on cell type annotation methods like Seurat v4 and Cell2location. However, SpaDo has demonstrated robustness to these methods (Fig. 3c, d). Moreover, advancements in more accurate cell type annotation and spot deconvolution methods could potentially enhance SpaDo's performance.”

2. The regional composition (SPACE) can capture diversity to some extent, but it shares similarities with existing spatial embedding methods, such as many spatial graph approaches in the field. I'm hesitant to conclude that this embedding surpasses the efficacy of graph neural network approaches that utilize high-dimensional gene expressions as input, as opposed to only cell type percentages (which may represent just a few dimensions). I am particularly concerned that this approach may oversmooth features and diminish tissue heterogeneity.

Response:

Thank you for your insightful comment, and we appreciate your concerns. We would like to provide additional clarification and analysis on the unique aspects and advantages of SpaDo, particularly, we would like to highlight the advantages of SPACE embedding comparing to other GNN-based embedding. The SPACE embedding, although simply designed, hold the following three advantages compared to other complex GNN-based spatial embedding methods like SEDR, SpaGCN, and STAGATE:

1.Interpretability: SPACE demonstrates good interpretability by aligning well with the biological characteristics of spatial domains. It operates on the hypothesis that cells or spots within the same spatial domain exhibit comparable spatially adjacent cell type proportions, and vice versa, indicating specific cell types with functional relationships.

2.Batch Effects Tolerance: SPACE effectively addresses batch effects by being designed based on spatial relationships of cell types, which is tolerant to batch effect. This design ensures consistency across slices through various automated cell type annotation strategies and clustering methods. We made this point clearly in our revised manuscript shown in Fig. 4 and Fig. S9.

3.Robustness and Noise Tolerance: Differences between different spatial domains are relatively obvious and do not require particularly fine spatial information to differentiate them. A recent study ("Lin S, Zhao Y, Yuan Z: Spatially Resolved Gene Expression is Not Necessary for Identifying Spatial Domains. bioRxiv 2023:2023.2010.2015.562443") has reached a similar conclusion. Therefore, although SPACE may oversmooth features and diminish tissue heterogeneity to some extent, it still performs well for the task of cross-slice spatial domain analysis. Additionally, SPACE exhibits strong robustness and tolerance to high noise (Fig. 3g, h).

Collectively, we think SPACE keeps a balance between the robustness and feature smoothing of the embedding in terms of spatial domain detection. We make these points clearly on page 4 and in the discussion section on page 15 of the revised manuscript (shown below).

Page 4:

“Particularly, we would like to highlight the advantages of SPACE embedding comparing to other complex GNN-based spatial embedding methods like SEDR, SpaGCN, and STAGATE: (1) SPACE is highly interpretable, aligning well with the biological nature of spatial domains, which often encompass specific cell types with functional relationships[9, 13, 21, 28, 29]. (2) SPACE effectively addresses batch effects, as it is designed based on the spatial relationships of cell types, ensuring consistency across slices through various automated cell type annotation strategies[24, 25, 27, 30] and clustering methods[15, 26]. As a result, SPACE is naturally suited for integration between multiple slices without the need for additional batch correction. (3) SPACE primarily relies on cell type annotations rather than detailed spatial information. The differences between spatial domains are apparent and do not require fine details, as supported by a recent study[31]. This characteristic makes SPACE tolerant to noise and robust to diverse variations in spatial transcriptomics data. Although SPACE may over smooth features and reduce tissue heterogeneity, it still performs well in cross-slice spatial domain analysis. Overall, SPACE keeps a balance between robustness and feature smoothing in spatial domain detection.”

Page 15:

“The key innovation of SpaDo lies in its design of a simple yet effective embedding called SPACE. By combining cell type annotation with spatial niche, SPACE successfully integrates gene expression and spatial information across multiple slices, demonstrating tolerance to high noise and batch effects (Fig. 3, 4). We highlight three main reasons why SPACE outperforms other complex spatial embedding methods like SEDR, SpaGCN, and STAGATE: (1) SPACE demonstrates good interpretability by aligning well with the biological characteristics of spatial domains. It operates on the hypothesis that cells or spots within the same spatial domain showcase comparable spatially adjacent cell type proportions, and vice versa, indicating specific cell types with functional relationships[9, 13, 21, 28, 29]. (2) SPACE effectively addresses batch effects, as it is designed by spatial relationships of cell types, ensuring consistency across slices through various automated cell type annotation strategies[24, 25, 27, 30] and clustering methods[15, 26]. (3) Differences between distinct spatial domains are relatively obvious and do not require particularly fine spatial information to distinguish them[31]. Therefore, although SPACE may over smooth features and diminish tissue heterogeneity, it still works well for the task of cross-slice spatial domain analysis. Additionally, SPACE exhibits strong robustness and tolerance to high noise (Fig. 3g, h). As a result, SPACE is naturally suited for integration between multiple slices. ”

3. I don't perceive this approach as a multi-slice technique. It concatenates representations from multiple slices (simply combining them along the sample dimension). It may be worthwhile to explore features that directly address the multi- slice issue, rather than relying on a concatenation approach. Any representation approaches can handle mutli-slice issue in this way.

Response:

Thank you for this valuable comment. We appreciate your insights, and we would like to address your concerns regarding the multi-slice nature of SpaDo.

Firstly, from a theoretical standpoint, SPACE is derived by fusing cell type annotations with the niche information of each cell/spot, achieving the integration of gene expression and spatial information. It is designed based on the spatial relationships of cell types, ensuring consistency across slices through various automated cell type annotation strategies and clustering methods. As a result, SPACE is naturally suited for integration between multiple slices without the need for additional batch correction. In essence, SPACE serves as a simple yet effective embedding strategy for both single ang multiple slices.

Secondly, to test if “Any representation approaches can handle mutli-slice issue in this way”, we performed additional analyses to assess the ability of SpaDo and other representation approaches, such as SEDR, SpaGCN, and STAGATE, to address batch effects (Fig. 4 and Fig. S9) relying on a concatenation approach. The results indicate that only SpaDo can effectively handle batch effects. We clarify this observation by emphasizing that embeddings generated through alternative methods, such as SEDR, SpaGCN, and STAGATE, are restricted to distinct training spaces within individual data slices. As a result, embeddings from different slices produced by these methods do not align, even when applying batch correction techniques like harmony. In contrast, SPACE is inherently grounded in the spatial distribution of cell types.

We added this additional information and clarification in page 8-10 in the revised manuscript (shown below). We hope this addresses your concerns.

“ SpaDo effectively mitigates batch effects in multi-slice integration Most existing spatial transcriptome analysis methods are limited to single-slice spatial domain analysis, as they are unable to integrate gene expression and spatial information across multiple slices. However, with the advancement of spatial transcriptomics, it has become possible to obtain multiple slices from the same tissue and integrate them to gain novel biological insights[20, 21]. Notably, one of the most significant challenges lies batch effect during the integration process. As mentioned earlier, SpaDo utilizes SPACE to integrate gene expression and spatial information. SPACE is designed to leverage the spatial relationships among cell types, which ensures consistency across slices through various automated cell type annotation strategies[24, 25, 30] and spot deconvolution methods[15, 26]. Therefore, SpaDo is theoretically tolerance to batch effects.

To further prove this point, we made comprehensive comparisons between SpaDo and other existing methods for addressing batch effects on both single cell and spot resolution spatial transcriptomics data (Fig. 4 and Additional file 1: Fig. S9). For spot resolution spatial transcriptomics, we tested 4 DLPFC slices, which belong the same sample with the same 7 layers. We compared SPACE utilized in SpaDo (Fig. 4a), embeddings obtained by SEDR, and SpaGCN with and without harmony[37] to correct batch effects (Fig. 4c). The results demonstrate that SpaDo's spatial embeddings (SPACEs) effectively mitigate batch effects across multiple slices. In contrast, the embeddings from SEDR and SpaGCN, even when combined with harmony[37], do not align well among multiple slices. Furthermore, we performed multi-slice domain detection with SpaDo (Fig. 4c, d). Then, for embeddings obtained by SEDR and SpaGCN combined harmony, we adopted similar strategy to perform multi-slice domain detection (Fig. 4e, f and see Methods). We can see that only SpaDo obtained consistent domain results between 4 DLPFC slices (Fig. 4d-f). We obtained the similar conclusions on 3 MERFISH data (Additional file 1: Fig. S9).

We clarify this observation by emphasizing that embeddings generated through alternative methods, such as SEDR, SpaGCN, and STAGATE, are restricted to distinct training spaces within individual data slices. As a result, embeddings from different slices produced by these methods do not align, even when applying batch correction techniques like harmony. In contrast, SPACE is inherently grounded in the spatial distribution of cell types, a feature that remains uniform across diverse slices. The good interpretability of SPACE in representing spatial domains, combined with this consistency, forms the foundation for its superior performance. ”

4. Clarification is needed on how hierarchical clustering cutoffs are determined.

Response:

Thank you for your valuable comment. In response to your concern, we have added an additional section in the methods part titled "The selection of proper domain numbers" to clarify how hierarchical clustering cutoffs are determined. You can find this information on pages 19-20 in the revised manuscript (shown below):

“The selection of proper domain numbers The spatial domain with different resolutions can be obtained by selecting proper domain numbers (Additional file 1: Fig. S1). To determine the optimal spatial domain number, SpaDo offers three optional strategies: (1) Automatic selection using the cutreeDynamic() function with parameter “deepSplit=2” from R package dynamicTreeCut; (2) Manually set by users based on their prior knowledge or specific requirements; (3) Visualization of the hierarchical trees and UMAP clustering results can assist in determining the optimal spatial domain number. The last two strategies allow for customization, providing a high level of flexibility and interpretability in the analysis of spatial domains. In this study, for each test data, different approaches were employed. If region labels were provided in the original study, the number of regions will be used as the spatial domain number. If not, SpaDo adopted the first strategy to determine the optimal spatial domain number.”

5. In the introduction: "However, these methods are restricted with high computational complexity, limited interpretability, and specific and strict assumptions." This statement appears unfair. Some of these methods are straightforward, while others have a solid statistical foundation.

Response:

Thanks for this valuable comments. We have revised the description in the introduction on page 2 in the revised manuscript (shown below).

“Nonetheless, challenges like relatively high computational complexity or limited interpretability may exist.”

6. "Although existing methods can detect spatial domains within a single tissue slice, they cannot directly handle cross-slice spatial domain analysis for multiple tissue slices in general." With the application of batch removal, concatenation, and clustering, most existing approaches can indeed be adapted for multi-slice tasks.

Response:

Thank you for your comment. This comment is similar to comment 3 shown above. We agree that existing approaches can be adapted for multi-slice tasks through the application of batch removal, concatenation, and clustering. Therefore, in the revised manuscript, we have addressed this by comparing SEDR, SpaGCN, and STAGATE, which are designed for single-slice domain detection, both with and without batch effect correction by harmony (Fig. 4 and Fig. S9 shown above). The results demonstrate that only SpaDo effectively addresses batch effects, providing additional evidence to support our claims.

7.Figure 2: The visualization of hierarchical clustering operation lacks significance here, as it is a standard approach.

Response:

Thank you for your suggestion. In response, we have revised the visualization of hierarchical clustering operation, moving it to Supplementary Figure S1 in the revised manuscript.

8.Supplementary Figure 4: More detailed annotations, including those pertaining to glutamatergic neural layers (as shown in original paper), could enhance the utility of this figure.

Response:

Thank you for your comment. We appreciate your suggestion for more detailed annotations in Supplementary Figure 4. However, we didn’t find information of manually annotated glutamatergic neural layers in the data from original paper (“https://github.com/CaiGroup/seqFISH-PLUS/blob/master/sourcedata.zip”).

9. Overall, the authors conducted experiments on numerous datasets; however, I find that the proposed approach did not address the multi-slice issue and may not introduce sufficient innovation for Genome Biology.

Response:

Thank you for your comment. We appreciate your perspective. In response to your comment, we would like to emphasize that SpaDo is designed to address the multi- slice spatial domain analysis, as demonstrated in our experiments and comparisons with other methods across multiple datasets. While we respect your viewpoint, we believe that the innovative aspects of SpaDo, particularly its SPACE embedding and effective handling of batch effects, contribute to advancing the field of spatial transcriptomics. We have worked to improve the presentation of our methodology and results in our revised manuscript.

Reviewer #3:

Thank you for your insightful comments. In the revised manuscript, we've clarified the resolution levels in Fig. 3, performed sensitivity tests on different cell type deconvolution methods, addressed concerns about batch effects, conducted ablation studies on the impact of multiple slices, and explained the distinction in strategies for obtaining cell type annotations. We believe these adjustments adequately address your concerns, and we remain open to further discussion or adjustments based on your feedback.

Below are point-to-point responses to each of your comments:

1. SpaDo detects domains at multiple levels of resolution. It's unclear which level of resolution was used for the evaluation in Fig.3. In addition, the other methods, e.g., Seurat, Scanpy, SpaGCN can also detect domains at different resolution by tuning the clustering resolution parameters. It's questionable whether the other methods achieved their best performance, as it seems that the authors didn't tune the resolution parameters for those methods.

Response:

Thank you for your valuable comment. We appreciate your observation regarding the resolution levels in Fig. 3. To clarify, in the initial manuscript, we utilized the minimum domain number at which SpaDo can detect TLS-like domains. This choice was made to highlight SpaDo's maximum sensitivity in comparison to other methods. We acknowledge the concern raised by the reviewer regarding the ability of "Seurat, Scanpy, SpaGCN" to detect domains at different resolutions through the tuning of clustering parameters. In response to this point, we conducted additional evaluations in the initial manuscript, specifically varying the number of domains. As we can see in Additional file 1: Fig. S8 in our revised manuscript, SpaDo consistently outperformed other methods even when a larger number of domains (domain number = 10) was used. We have incorporated these clarifications in our revised manuscript on page 6.

2. SpaDo uses cell2location as the starting point for spot resolution data. However, different cell type deconvolution methods usually generate results that are inconsistent with each other. It's important to know how sensitive is SpaDo to different cell type deconvolution methods. Is cell2location always the best option for different datasets?

Response:

Thank you for this valuable comment. The choice of Cell2location as the starting point for SpaDo is grounded in its strong performance, as demonstrated in a third-party benchmarking paper [Li B, Zhang W, Guo C, Xu H, Li L, Fang M, Hu Y, Zhang X, Yao X, Tang M, et al: Benchmarking spatial and single-cell transcriptomics integration methods for transcript distribution prediction and cell type deconvolution. Nat Methods 2022, 19:662-670.].

In addition, we have conducted a sensitivity test to explore the use of other widely employed spot deconvolution methods, such as SPOTlight and RCTD, across 12 spot resolution DLPFC dataset (Fig. 3d) in the revised manuscript. The results indicated that SpaDo exhibits robustness to various spot deconvolution methods, with Cell2location consistently achieving the highest median performance. These additional insights have been incorporated into our revised manuscript on pages 7 (shown below).

“The Cell2location was selected based on its strong performance in a third-party benchmarking paper [34]. Furthermore, we validated the robustness of SpaDo to Seurat v4 parameters for cell type annotation across three single-cell spatial transcriptomics datasets (Fig. 3c). We also investigated the application of other widely used spot deconvolution methods, including SPOTlight[35] and RCTD[36], for 12 spot resolution DLPFC slices (Fig. 3d). We can see that SpaDo is robust to spot deconvolution methods, with Cell2location achieving the highest median.”

3. There are usually batch effects for different slices, especially for those sampled from different individuals. However, the authors didn't discuss the batch effects in different slices and there is not a clear procedure of removing batch effects. The authors should discuss how the batch effect was handled in SpaDo and how could it possibly influence the performance of SpaDo.

Response:

Thank you for your valuable comment. In our revised manuscript, we conducted a comprehensive comparison between SpaDo and other existing methods for addressing batch effects in both single-cell and spot resolution spatial transcriptomics data (Fig. 4 and Additional File 1: Fig. S9). Specifically, for spot resolution spatial transcriptomics, we tested four DLPFC slices, each with the same seven layers. We compared the performance of SPACE utilized in SpaDo (Fig. 4a) with the embeddings obtained by SEDR and SpaGCN, both with and without harmony to correct batch effects (Fig. 4c). The results clearly demonstrate SpaDo's effectiveness in removing batch effects, whereas SEDR and SpaGCN exhibit significant batch effects even when combined with harmony.

Furthermore, we conducted multi-slice domain detection with SpaDo (Fig. 4c, d). For embeddings obtained by combining harmony with SEDR and SpaGCN, we employed a similar strategy for multi-slice domain detection (Fig. 4e, f). The results highlight that only SpaDo consistently obtains domain results across the four DLPFC slices (Fig. 4d- f). Similar conclusions were obtained from our analysis of three MERFISH datasets (Additional File 1: Fig. S9). We appreciate your attention to this crucial aspect, and these findings underscore SpaDo's efficacy in addressing batch effects across different slices. Further details and clarifications have been added to pages 9-10 of the revised manuscript (shown below).

“ SpaDo effectively mitigates batch effects in multi-slice integration Most existing spatial transcriptome analysis methods are limited to single-slice spatial domain analysis, as they are unable to integrate gene expression and spatial information across multiple slices. However, with the advancement of spatial transcriptomics, it has become possible to obtain multiple slices from the same tissue and integrate them to gain novel biological insights[20, 21]. Notably, one of the most significant challenges lies batch effect during the integration process. As mentioned earlier, SpaDo utilizes SPACE to integrate gene expression and spatial information. SPACE is designed to leverage the spatial relationships among cell types, which ensures consistency across slices through various automated cell type annotation strategies[24, 25, 30] and spot deconvolution methods[15, 26]. Therefore, SpaDo is theoretically tolerance to batch effects. To further prove this point, we made comprehensive comparisons between SpaDo and other existing methods for addressing batch effects on both single cell and spot resolution spatial transcriptomics data (Fig. 4 and Additional file 1: Fig. S9). For spot resolution spatial transcriptomics, we tested 4 DLPFC slices, which belong the same sample with the same 7 layers. We compared SPACE utilized in SpaDo (Fig. 4a), embeddings obtained by SEDR, and SpaGCN with and without harmony[37] to correct batch effects (Fig. 4c). The results demonstrate that SpaDo's spatial embeddings (SPACEs) effectively mitigate batch effects across multiple slices. In contrast, the embeddings from SEDR and SpaGCN, even when combined with harmony[37], do not align well among multiple slices. Furthermore, we performed multi-slice domain detection with SpaDo (Fig. 4c, d). Then, for embeddings obtained by SEDR and SpaGCN combined harmony, we adopted similar strategy to perform multi-slice domain detection (Fig. 4e, f and see Methods). We can see that only SpaDo obtained consistent domain results between 4 DLPFC slices (Fig. 4d-f). We obtained the similar conclusions on 3 MERFISH data (Additional file 1: Fig. S9).

We clarify this observation by emphasizing that embeddings generated through alternative methods, such as SEDR, SpaGCN, and STAGATE, are restricted to distinct training spaces within individual data slices. As a result, embeddings from different slices produced by these methods do not align, even when applying batch correction techniques like harmony. In contrast, SPACE is inherently grounded in the spatial distribution of cell types, a feature that remains uniform across diverse slices. The good interpretability of SPACE in representing spatial domains, combined with this consistency, forms the foundation for its superior performance. ”

4. The most important contribution of SpaDo is its improved accuracy in detecting spatial domains by integrating multiple slices. However, it is unclear that how much the multiple slices contribute to the accuracy comparing to using single slice. The authors should perform ablation studies that use SpaDo on each single slice and compare with the results from combining multiple slices.

Response:

Thank you for this comment. While we believe that the key contribution lies in the design of SPACE as the unified spatial embedding, enabling cross-slice spatial domain detection, reference-based annotation, and cross-slice clustering, we acknowledge the significance of demonstrating the impact of multiple slices on accuracy. In response to your suggestion, we have included an ablation study using DLPFC_151675 and DLPFC_151676 datasets as examples. This study clearly illustrates SpaDo's significant improvement in performance when integrating multiple slices (Additional file 1: Fig. S7). The results underscore SpaDo's adaptability and effectiveness, particularly in challenging dataset scenarios. You can find the detailed findings in Fig. S7 and in page 5 of our revised manuscript (shown below).

“In addition, using two DLPFC datasets (DLPFC_151675 and DLPFC_151676) as examples, we demonstrated SpaDo's ability to significantly improve its performance by integrating multiple slices (Additional file 1: Fig. S7). This underscored SpaDo's adaptability and effectiveness in optimizing results, especially in challenging dataset scenarios.”

5. When using Seurat clustering as the cell type annotation, SpaDo is basically a spatial smoothing of the Seurat cell clusters. However, in Fig. 3e, SpaDo detects domains where no clustering signal could be observed from the Seurat domains. The authors should discuss why SpaDo significantly outperformed Seurat when using it as the starting point.

Response:

Thank you for your comment. It's essential to clarify that SpaDo employs different strategies for obtaining cell type annotations based on the resolution of the spatial transcriptomic data.

For single-cell resolution spatial transcriptomics data, SpaDo utilizes Seurat as the starting point to obtain cell type annotations.

On the other hand, for spot resolution spatial transcriptomics data, such as the datasets tested in Fig. 3e the reviewer concerned (Fig. 2e in our revised manuscript), SpaDo uses deconvolution method Cell2location instead of Seurat for acquiring cell type annotations. This distinction is crucial in understanding the methodology used in our analysis.

**2^nd^ round**

**Reviewer 1**

The authors have very effectively addressed comments.

**Reviewer 2**

I appreciate authors' efforts addressing my questions. I am still not convinced by the novelty of the approach. I still have the following concerns:
1. "However, SpaDo has demonstrated robustness to these methods (Fig. 3c, d). Moreover, advancements in more accurate cell type annotation and spot deconvolution methods could potentially enhance SpaDo's performance." Theses two sentences are contradictory with each other. If SpaDo is robust to these methods, why it can benefit from advancements in new convolution methods?
2. For the benefits from SPACE embedding, I only agree with its interpretability. All other approaches can handle the rest. My concern is this smooth operation will not improve the identification of heterogeneous tissue structure -- it will more likely to produce simpler tissue structure.
I will not take cross-slice ability equivalent to batch-removal ability. The additional information from cross-slice is in its 3D spatial relation.

**Reviewer 3**

The authors' response has addressed all the questions I raised before. I have no more request.

**Authors’ response**

Reviewer #2:

1. "However, SpaDo has demonstrated robustness to these methods (Fig. 3c, d). Moreover, advancements in more accurate cell type annotation and spot deconvolution methods could potentially enhance SpaDo's performance." Theses two sentences are contradictory with each other. If SpaDo is robust to these methods, why it can benefit from advancements in new convolution methods?

Response:

Thank you for this valuable comment. We demonstrate the robustness of SpaDo to various cell type annotation methods, implying that even in cases where the performance of cell type annotation and spot deconvolution methods is slightly suboptimal, SpaDo exhibits relatively favorable performance. It should be noted that this is not to suggest that the performance of SpaDo is unrelated to the accuracy of cell type annotation and spot deconvolution methods.

To mitigate potential misunderstandings, we revise the sentence as follows in page 15 of our revised manuscript: “Nevertheless, even in scenarios where cell type annotation methods exhibit slightly suboptimal performance, SpaDo continues to demonstrate relatively favorable outcomes (Fig. 3c, d).”

2. For the benefits from SPACE embedding, I only agree with its interpretability. All other approaches can handle the rest. My concern is this smooth operation will not improve the identification of heterogeneous tissue structure -- it will more likely to produce simpler tissue structure. I will not take cross-slice ability equivalent to batch- removal ability. The additional information from cross-slice is in its 3D spatial relation.

Response:

Thank you for these comments.

Firstly, we appreciate your acknowledgment of the interpretability of our SPACE embedding.

Secondly, we fully understand the reviewer's concern that the smooth operation in SpaDo might lead to a tendency to discover simpler spatial structures. However, through our analysis of over 40 spatial transcriptomic datasets, we have not observed such a tendency. To further demonstrate that the smooth operation employed by SpaDo may not predispose it to discover simpler spatial structures, we present the following three additional examples.

1.In Figure S4a (shown below), "Layer 2-3 lateral" and "Layer 2-3 medial" exhibit finely detailed and similar spatial structures that are challenging to distinguish, given their shared layer 2-3 classification. SpaDo effectively identifies these structures, while other methods failed (Fig. S4b).

2.As depicted in Figure S6a (shown below), "HPC" represents an intricately detailed spatial structure with relatively fewer cells, posing a detection challenge. SpaDo successfully identifies "HPC," whereas alternative methods either fail to detect it or cannot differentiate between "HPC" and "L5" (Fig. S6b).

3."TLS" is a heterogeneous tissue structure with limited cell representation, making it difficult to detect. SpaDo outperforms other methods in TLS detection (Fig. 2e).

We agreed with the reviewer that such a discovery maybe counterintuitive, however, we speculated that three reasons may exist to explain it:

Firstly, although SpaDo employs smooth operation and may result in the loss of spatial information for individual cells, however, for the entire spatial domain, the most crucial spatial information, namely the composition of cell types in space, is adequately preserved.

Secondly, smooth operation inherently possesses robust noise resistance capabilities, facilitating the elimination of noise in spatial transcriptomic data in the domain identification.

Thirdly, the distinctions between spatial domains are evident and do not necessitate fine details, as supported by a recent study [Lin S, Zhao Y, Yuan Z: Spatially Resolved Gene Expression is Not Necessary for Identifying Spatial Domains. bioRxiv 2023:2023.2010.2015.562443.].

We have incorporated these points into page 15 of our revised manuscript as follows:

“Therefore, although SPACE may introduce some feature smoothing and reduce tissue heterogeneity, it still demonstrates strong performance in multi-slice spatial domain analysis. In our study, we did not observe that SPACE simplifies spatial structures (Fig. S4, S6, and Fig. 2e). We speculate that three reasons may exist here. Firstly, while there may be a loss of spatial information at the individual cell level, the essential spatial information—specifically, the composition of cell types throughout the entire spatial domain—is effectively preserved by using SPACE embedding. Secondly, the inherent noise resistance of smooth operation aids in eliminating noise from spatial transcriptomic data. Lastly, recent study [31] support that the discernible distinctions between spatial domains do not necessitate intricate details. In summary, SPACE maintains a delicate balance between noise tolerance and feature smoothing in spatial domain detection, making it well-suited for the integration of multiple slices.”

Thirdly, we acknowledge the reviewer's distinction between cross-slice ability and batch-removal ability. To address this, in the revised manuscript, we use “multi-slice” instead of “cross-slice” to describe SpaDo.
